# Graphical Fourier-coefficient analysis as a paper-based method for teaching structure factors

**DOI:** 10.1107/S2056989026000745

**Published:** 2026-01-29

**Authors:** Thomas E. Weirich

**Affiliations:** a RWTH Aachen University, Gemeinschaftslabor für Elektronenmikroskopie (GFE), Ahornstr. 55, D-52074 Aachen, Germany; Harvard University, USA

**Keywords:** structure factor, discrete Fourier analysis, X-ray diffraction, teaching

## Abstract

The structure factor is a fundamental concept in X-ray crystallography, yet it is often introduced via formal sum expressions that make it difficult to realize its connection to the atomic structure. Therefore, a one-dimensional Fourier analysis method is proposed as an alternative approach for teaching. The method is a graphical discrete Fourier analysis-based approach that is used to determine the cosine and sine coefficients, *A* and *B*, respectively, of the structure factor from projections. To allow seamless and easy integration into existing curriculums, worksheets with the corresponding function graphs for determining the coefficients are provided. The application of the method is demonstrated by several examples, which illustrate its use for enhancing the understanding between structure and systematic extinctions and existing constraints for structure factor phases. Although this approach has not been developed for crystal structure analysis, it does provide the user with an intuitive framework for developing a conceptual understanding between the structure factors and atomic arrangement in crystalline materials.

## Abstract 2

A one-dimensional graphical Fourier analysis is proposed as a teaching approach to visualize the cosine and sine coefficients of structure factors and to provide a visual illustration of the relationship between atomic arrangement, symmetry and diffraction patterns.

## Introduction

In X-ray crystallography, the structure factor 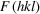 in equation 1 is a central figure as it describes the positions and scattering properties of atoms within a unit cell in terms of the amplitude 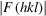 and phase 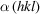 of the diffracted X-rays (see for example Buerger, 1970 ▸, Glusker & Trueblood, 1985 ▸, Ladd & Palmer, 2003 ▸).
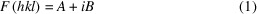
with
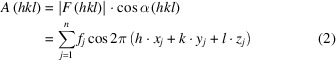
and
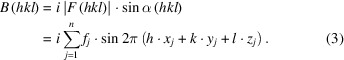
Herein, 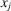, 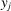, and 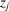 are the relative (fractional) coordinates of atom *j* along the axes of the unit cell *a*, *b*, and *c*, 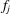 is the scattering amplitude of the atom *j*, and *hkl* denote the Laue indices of a particular reflection. The phase angle of the corresponding structure factor amplitude is given by equation (4) and the magnitude can be calculated from equation (5) (Buerger, 1970 ▸).
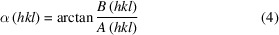

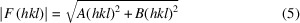
In an experiment, the (unscaled and uncorrected) amplitude of the structure factor can be obtained by taking the square root of the intensity; see for example Stout & Jensen (1989 ▸) page 178, eq. (7.1) Combined with the corresponding phase information, the structure-factor amplitudes allow the three-dimensional electron-density distribution within the unit cell to be calculated (Bragg, 1915 ▸, p. 270; Woolfson, 2018 ▸), from which the atomic arrangement of the crystal can be derived. Given its importance, the structure factor is a standard topic in crystallography and diffraction-related courses in chemistry, materials science, and physics. The significance of this subject is underscored not only by its coverage in textbooks, but also by the existence of a dedicated IUCr teaching pamphlet on it (Wallwork, 1980 ▸).

However, students often find it difficult to make a connection between the structure factor as presented by equations and the underlying arrangement of atoms in the unit cell of a crystal. As a result, to many, the subsequently described process of structural analysis remains merely a black box method for experts who deal with some not fully understood formulas.

The subsequent example illustrates and clarifies the potential didactic challenges that exist. Suppose that the aim is to explain the effect of the structure factor on the extinction rules for plane group *pg* (No. 4) with atom positions 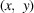 and 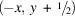. Using the structure-factor equations given above and just focusing on the geometric parts, this readily yields the following expressions for 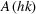 and 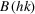:
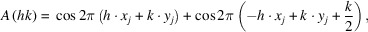

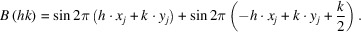
The crux of the issue is evident, as it is unreasonable to expect students to identify the general reflection condition 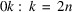 from these equations. A look at older volumes of the International Tables is not helpful either, as the geometric structure-factor formulas listed there were developed for efficient computing and are just a rearrangement of the above equations, but are likewise difficult to interpret (International Tables for Crystallography, 1969 ▸, p. 369).
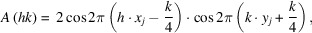

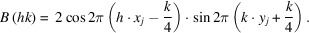
The final fallback for a (brute-force) interpretation would then be to enter the equations into a spreadsheet program and experiment with different values for the indices, to empirically verify the reflection conditions specified in *International Tables*. In summary, this treatment of the matter is not very effective as it only offers students little insight.

In order to bridge this gap between the formal language of equations and their significance for the practical application of X-ray diffraction, a graphical Fourier-coefficient analysis is presented below, which was developed for teaching purposes several years ago. This approach is an extension of the concepts summarized earlier (Weirich, 2006 ▸), and it has been designed to be visual and intuitive, thus making it especially suitable for those new to X-ray crystallography.

## Graphical Fourier-coefficient analysis

**Figure 1:**
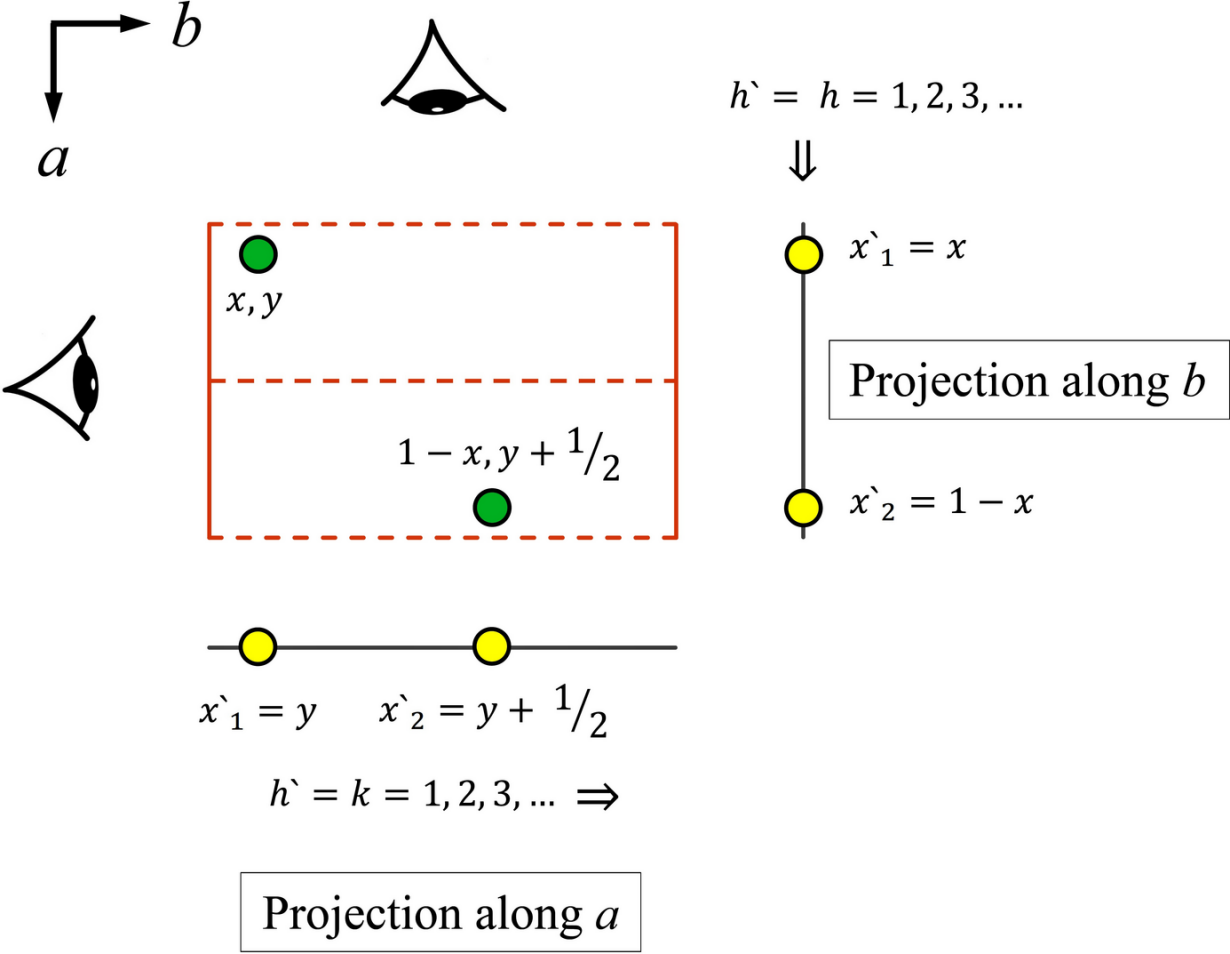
Illustration of the projection procedure described in the main text. For plane group symmetry *pg* (International Tables, No. 4) the two shown atom positions are related by a glide line *g* (- - -) normal to the *a* axis. To allow the analysis of the extinction rules along the two principal axes in one dimension, the atoms must be projected onto lines as shown. This yields a point symmetric atom distribution on the line for the projection along the *b* axis and a non-symmetrical atom distribution for the projection along the *a* axis. In the one-dimensional models, the cosine and sine Fourier waves of order *h*′ will pass the one-dimensional unit cell in the direction of the arrows. A representation that shows a set of 3rd order cosine waves that traverse the unit cell is given in Fig. 2 ▸. Thus, the projection along the *b* axis is used to study the extinctions along the *a* axis and *vice versa*.

**Figure 2:**
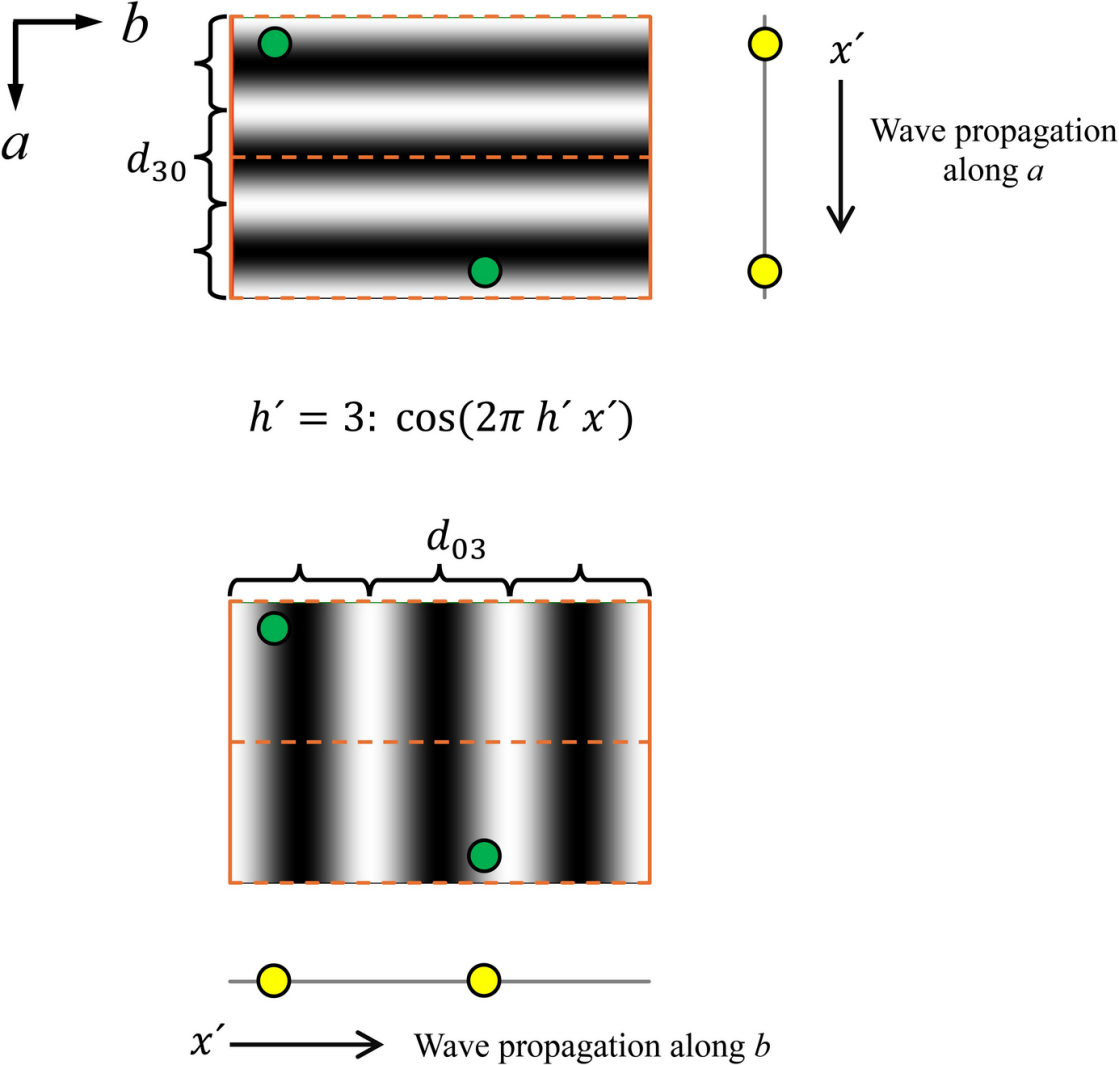
Two sets of 3rd order cosine waves (*h*′ = 3) that cross the unit cell of plane group *pg* along the principal axes *a* and *b*. Note that the order determines the number of full Fourier wave trains with wavelength *d*_*h*′_ = *a*,*b*/*h*′ along the line of projection. Moreover, the illustration shows that the plane waves cannot sense the position of an atom along the direction of projection (see Fig. 1 ▸). This feature is essential as it allows the use of line projections for the calculations with equations 6 and 7.

The example above can be resolved more elegantly if it is realised that the structure-factor expressions in equations 2 and 3 are the result of the discrete Fourier transform of the crystal structure. Readers who are less familiar with discrete Fourier transforms are referred to the book by J. F. James as an introduction to Fourier transforms (James, 1995 ▸). For pedagogical reasons, it has been proven advantageous to break down the three-dimensional case to one dimension, as this allows the relationships to be shown by simple plots. Accordingly, equations (2) and (3) are:
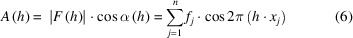
and
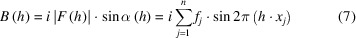
The working principle of a discrete Fourier transform applied on a one-dimensional object function 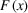 is described in simple terms to show the similarity with the latter formulas. A discrete Fourier transform can be carried out graphically by plotting the function 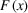 on graphs of 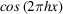 and 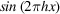 for different orders of *h* (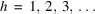). The range of *x* is a fractional value between zero and one, which covers the full range of the available data along the *x* axis. Within the actual calculation of the Fourier transform, the value of 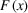 is multiplied with the value of the cosine (or sine) function at the same position *x*. The resultant values are finally summed to yield the value of coefficients 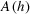 for the cosine and 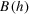 for the sine part. This concept can be directly transferred to our case if we assume a linear structure with *j* atoms at the positions 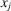. The corresponding *y* value at the position 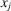 of every atom is given by the scattering amplitude 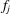 of the atom. In contrast to a standard (continuous) mathematical function, our model has the characteristics that the function values 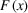 are equal to zero everywhere except at the positions 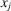 where the function value takes the value 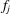. Our model could therefore also be interpreted as a function composed of several Dirac delta functions with peak heights 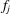. For this reason, it is reasonable to sum up the amplitude values only at the positions 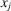 of the atoms, since all other values are zero. This is exactly the behaviour that is described by equations (6) and (7) as a formula. However, real crystal structures are three-dimensional, and it is therefore required to link them with the one-dimensional model presented here. The key that allows this is that each set of the cosine and sine Fourier waves in equations (2) and (3) propagates normal to the lattice planes *hkl* through the unit cell [note that the Fourier wavelength is always equal to the lattice spacing 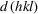]. In our one-dimensional model, the sine and cosine components of the Fourier waves are restricted and can only propagate along the *x* axis. Thus, the unit cell must be oriented such that the wave propagation in three dimensions coincides with the direction of wave propagation in one dimension. This is achieved by aligning the lattice planes 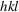 so that they are perpendicular to the direction of propagation of the Fourier wave in one dimension, allowing the three-dimensional atomic positions to be projected onto the *x* axis. For illustration, the projection procedure is shown for the earlier mentioned plane group *pg* in Fig. 1 ▸. Moreover, Fig. 2 ▸ shows that the plane waves cannot detect different atom positions in the direction of projection. It is essential to understand that it is this feature that facilitates the use of line projections for calculations with equations (6) and (7).

**Figure 3:**
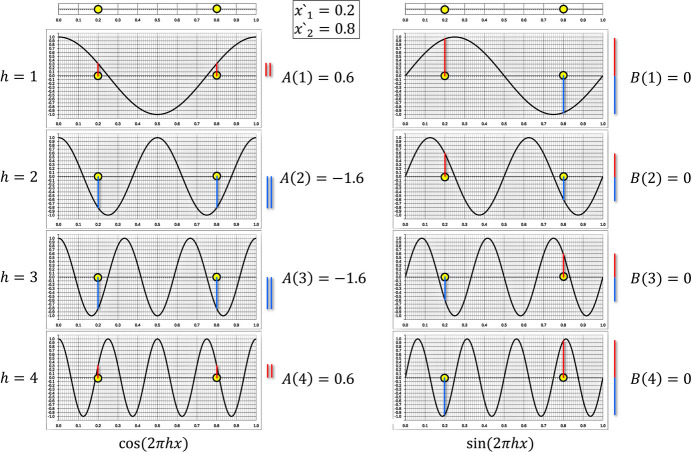
Fourier analysis plots for the projection of plane group *pg* along the *b* axis (see Fig. 1 ▸) with atom positions at *x*′_1_ = 0.2 and *x*′_2_ = 0.8 that allow investigation of the reflections along the *a* axis. The positions of the atoms were marked initially on the upper lines on each side and then (using a ruler) projected downwards into the graphs. In order to derive the contribution of each atom, it is only necessary to read the function value at the position of the atoms (positive and negative contributions are indicated by lines of different colours). After all atoms have been processed in each a plot, the individual contributions are summed to yield the values for 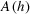 and 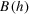, respectively. In agreement with plane group *pg*, no reflections exist with a structure-factor amplitude 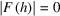 (use eq. 5). However, as all sine terms vanish due to the presence of the inversion centre (mirror point) in the one-dimensional unit cell, the structure-factor phases for the *h0* reflections in plane group *pg* are restricted to 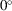 [the positive amplitudes 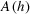] and 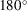 for the amplitudes with negative signs (use eq. 4).

**Figure 4:**
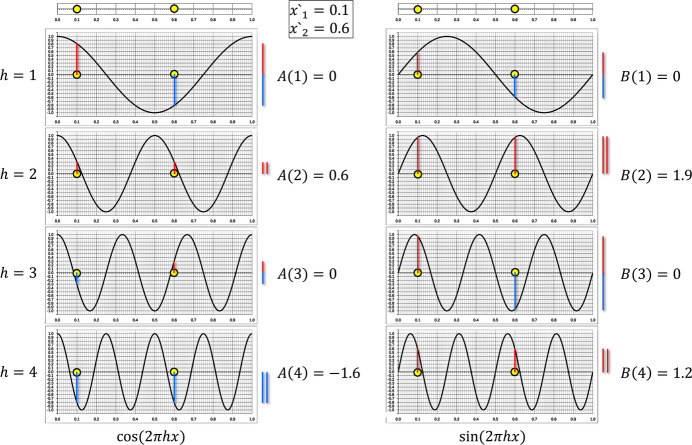
Fourier analysis plots for the projection of plane group *pg* along the *a* axis (see Fig. 1 ▸) with atom positions at *x*′_1_ = 0.1 and *x*′_2_ = 0.6 that allow investigation of the reflections along the *b* axis. The processing was performed in analogy to the description in Fig. 2 ▸. As the obtained amplitudes thereby are all zero for the odd numbers of *h*′, it can be concluded that the extinction conditions for plane group *pg* along the *b* axis are 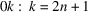, which is in full agreement with the corresponding reflection condition (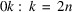) given in Volume A of International Tables for Crystallography. Moreover, it can be concluded the phase values for reflections with non-zero amplitude are not restricted since their *B* terms are not zero. Hence, the phase angles for *h* = 1 and 3 are zero because *A*(*h*) and *B*(*h*) are positive. According to equation 4 and the lookup table in Appendix A[App appa], *h* = 2 [+*A*(2), +*B*(2)] yields 72.5° for the structure-factor phase, and for *h* = 4 [−*A*(4), +*B*(4)] a phase value of 143.1° (= −36.9° + 180°) is obtained.

To perform the determination of the Fourier coefficients on paper, a set of sine and cosine plots was prepared for 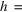 1 to 10, which only requires the positions of the atoms to be drawn (see supporting information). Figs. 3 ▸ and 4 ▸ show the corresponding plots for the two line projections of plane group *pg* with atom positions (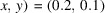 and (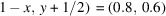. The evaluation of the plots in Fig. 3 ▸ (projection direction along *b* in Fig. 1 ▸) for the *h0* reflections along the *a* axis readily indicates no systematic absence of reflections. Moreover, it can be seen that the corresponding *B* coefficients are all zero, which is due to the point symmetrical distribution of the atoms along the one-dimensional unit cell. Therefore, it can be concluded that the structure factor phases for the *h0* reflections in plane group *pg* are confined to 0° and 180° (see Table 1 ▸ in Appendix A[App appa] for reference). However, an evaluation of the corresponding plots in Fig. 4 ▸ (projection direction along *a* in Fig. 1 ▸) for the *0k* reflections along the *b*axis does prove extinctions according to 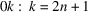, which agrees with the reference (International Tables for Crystallography, 1969 ▸). It is important to note that the amplitudes were determined entirely graphically but are in full agreement with the numerical values calculated using equations 6 and 7.

The subsequent examples will demonstrate the application of this method in the analysis of some more materials-oriented phenomena.

### Investigating effects caused by the scattering amplitude

In his seminal 1913 ▸ report on the first structural analysis using X-rays, W. L. Bragg demonstrated that rock salt (NaCl) and potassium chloride (KCl) both belong to the same cubic crystal system and share an identical atomic arrangement in the crystalline state. However, unlike NaCl, the X-ray diffraction pattern of KCl made it look like a simple primitive lattice and did not exhibit a prominent *111* diffraction peak (Bragg, 1913 ▸). A corresponding diffraction pattern of these measurements was later reproduced in books (Bragg & Bragg, 1915 ▸, p. 89; Bragg & Bragg, 1949 ▸, p. 40). However, in the 1913 ▸ study it was already identified that the different scattering power of atoms was the reason for the seemingly different lattices. In subsequent studies, it was then established that the scattering amplitude (the so-called *atom form factor*) depends strongly on the number of electrons of an atom and the diffraction angle (see ch. IX in Bragg & Bragg, 1949 ▸).

**Figure 5:**
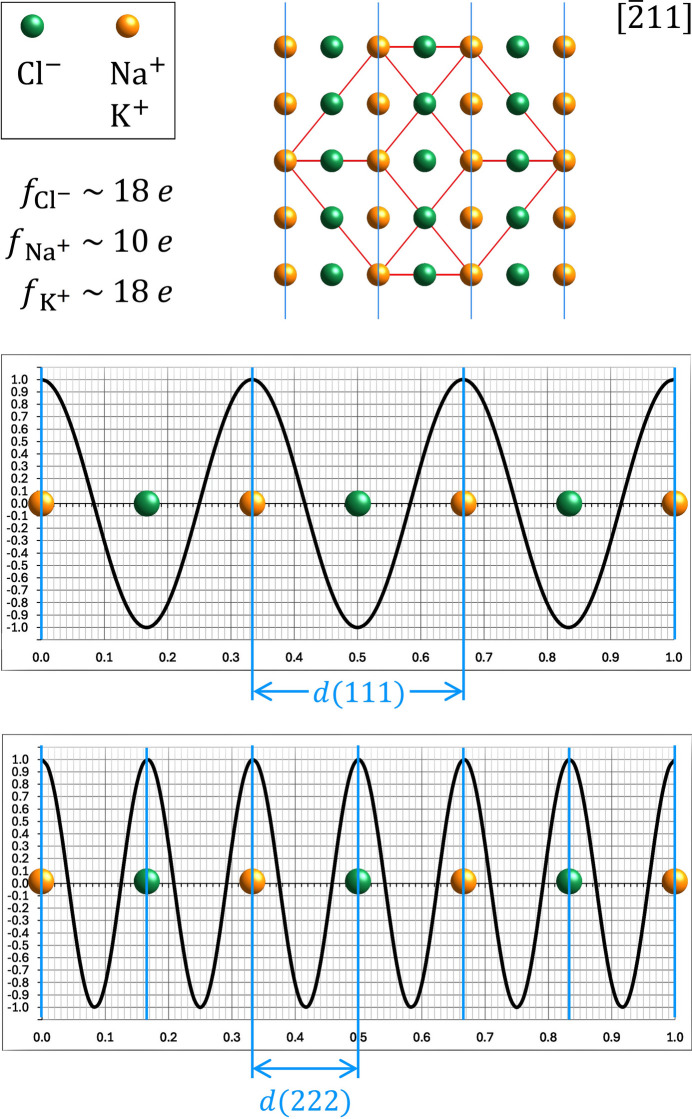
The Fourier analysis graph for the 111 reflection of the *MX* rock salt structure in the upper graph explains the finding of W. L. Bragg that this reflection was observed for NaCl, but not for KCl. As seen, all cations (orange) match with the positive maxima and all chlorine anions (dark green) coincide with the negative maxima. As *M*:*X* is 1:1 in this structure, it can be concluded that positive and negative contributions of *M* and *X* cancel each other out if the scattering amplitudes of the cations and anions have approximately the same magnitude (the same number of electrons in their shells). This is the case for KCl, where K^+^ and Cl^−^ are isoelectronic ions (see above figure). However, in the case of NaCl, the individual contributions of the ions are different, which leads to a net value for the *A* coefficient. This, in turn, results in the appearance of the 111 reflections in NaCl. As illustrated in the lower graph, for the higher-order 222 reflection all positive maxima are coincident with the positions of the ions, so this reflection is present in the diffractograms of NaCl and KCl.

**Figure 6:**
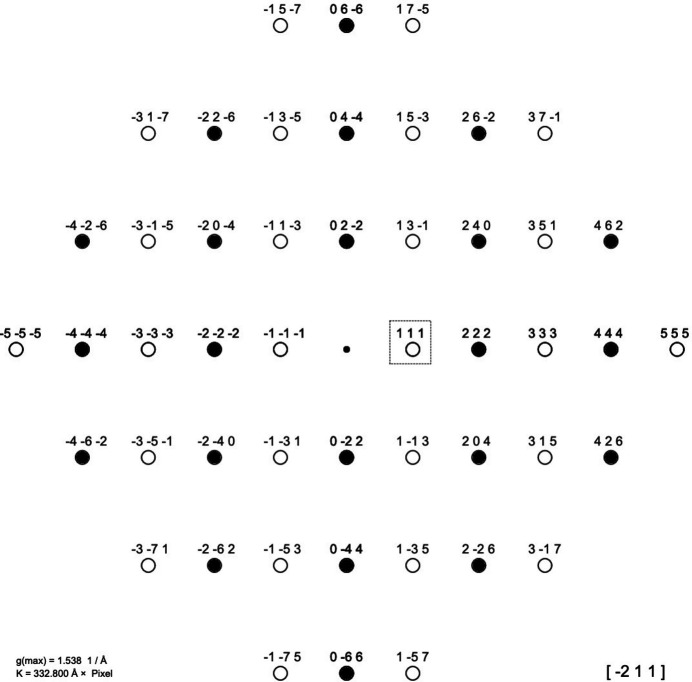
The figure shows a schematic [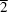
11] diffraction pattern for the *MX* rock salt structure. If the scattering factors of *M* and *X* differ, the open circles represent weak diffraction spots, as outlined in detail in Fig. 5 ▸ for the 111 reflection of NaCl boxed here. However, if the two scattering factors are of the same magnitude, which is the case for K^+^ and Cl− in potassium chloride (both ions carry 18 shell electrons), the scattering from each ion is just cancelled out by the scattering of the counter-ion. As a result, the intensities of all reflections with open circles are zero. The latter is the case for reflections in this pattern where all *hkl* have odd indices. However, the two ions are always in phase and add their scattering power to yield strong reflections when all *hkl* are even.

Using the one-dimensional Fourier analysis plots allows one to easily to follow the Braggs’ interpretation. Since a mere qualitative discussion is sufficient, it can be assumed that the scattering amplitudes of the involved atomic species are equal to their number of shell electrons. As shown in Fig. 5 ▸, the NaCl (KCl) unit cell was oriented along [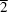
11], meaning that the 111 lattice planes are normal to the *x* axis of the line projections. A corresponding schematic zone-axis-aligned diffraction pattern for the projection along [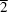
11] is shown in Fig. 6 ▸. In principle, it would have been enough to project just the atoms within one pair of the *111* lattice planes but, for clarity, this was done across the entire unit cell. Since the projection of the atoms is symmetrical with respect to the indicated *111* interplanar distance, all *B* coefficients must be zero (see Fig. 3 ▸). Therefore, only the cosine graphs require consideration in the subsequent discussion. From the set of prepared cosine functions provided in the supporting information, the one with 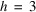 was chosen (see the upper graph in Fig. 5 ▸), as this matches the 111 interplanar distance 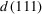. As can immediately be seen, all cations (Na^+^, K^+^) are located at the positive maxima of the cosine wave, while all anions (Cl^−^) are located at the negative maxima. Therefore, if the scattering amplitudes of the cations and the chlorine anions are equal, as is the case for K^+^, the contributions of both species will cancel each other out, resulting in a zero *A* coefficient and the absence of the 111 reflection. However, if the cation’s scattering amplitude is smaller than that of the chloride anion (as is the case for Na^+^), the net contribution will yield a negative *A* coefficient and the presence of a 111 reflection. As the *A* coefficient is negative and the *B* coefficient is zero, the corresponding phase of the structure factor is 180° (see Table 1 ▸ in Appendix A[App appa]). In agreement with the Braggs’ finding (Bragg & Bragg, 1949 ▸, p. 40), the corresponding analysis for the higher order 222 reflection (lower graph in Fig. 5 ▸) is found to be strong for KCl and NaCl since all positive maxima coincide with the positions of all atoms along the line.

**Figure 7:**
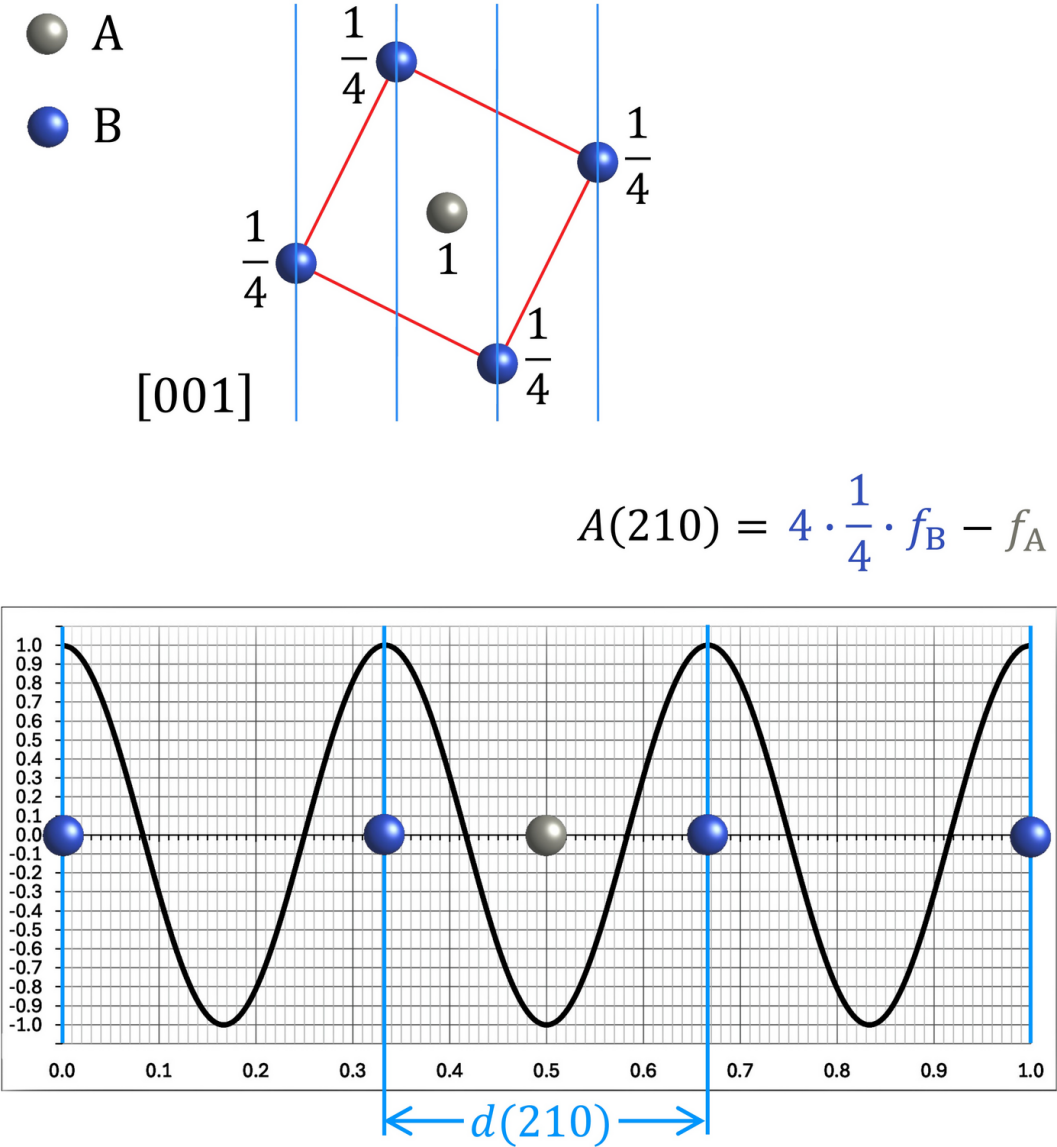
The above illustration shows how Fourier analysis graphs can be used to derive a general rule for the appearance of the 210 reflection in the ordered CsCl-type variant, and its disappearance in the disordered bcc-type variant. In this case, the entire contents of the unit cell must be projected onto the line, as shown. Note the point symmetry with the grey *B* atom at the centre, which means that all *B* coefficients after summation of the individual scattering amplitudes will be zero. As in the example shown in Fig. 3 ▸, therefore, only the *A* coefficient must be considered. As each of the *B* atoms (blue) shown represents two atoms in projection along the [001] direction, these must be counted twice. However, as each corner atom belongs to eight unit cells in the three-dimensional crystal structure, its contribution to the summation of the individual amplitudes must be weighted by the factor 1/8. Thus, the effective weighting factor for the *B* atoms is 1/8 × 2 = 1/4, as indicated. As the *A* atom at the centre of the structure is not shared with other unit cells, it is assigned a weighting factor of 1. As can now be seen for the 210 reflection, if all the atoms have the same scattering amplitude, as in the disordered bcc-type structure, the four blue *B* atoms with a positive contribution will cancel out the negative contribution from the one grey *A* atom in the centre due to the 180° phase difference between the two sets of atoms. If ordering takes place and the CsCl-type structure is formed, either the sum of the *B* atoms (= *f*_*B*_) will exceed the contribution of the *A* atom in the centre [*A*(210) is positive and the structure factor will be zero], or *vice versa* [*A*(210) is negative and the structure factor will be 180°, see look-up table in Appendix A[App appa]]. In both cases, the structure-factor amplitude will be non-zero, and a weak reflection will be observed in the diffraction pattern.

Similar effects that cause the presence or extinction of some reflection groups can also be observed in other material systems. The perhaps most well-known example is found in the Cu–Zn (brass) system, where an ordered distribution of atoms with cubic space-group symmetry *Pm*
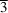
*m* (B2, CsCl type) and a disordered (high-temperature) form with cubic space-group symmetry *Im*
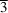
*m* (A2, W type) exist. Figs. 7 ▸ and 8 ▸ demonstrate this, showing a CsCl-type structure projected along the [001] direction, as well as the corresponding schematic diffraction pattern. The same two structure types are also found in the Co–Fe, Fe–Si, and Ni–Al systems. All these systems have in common that the ordered structures show, in addition to the bcc type reflections, reflections such as 100, 111, or 210. Using Fourier analysis graphs, the generation of additional reflections (in the ordered phase) or their extinction (in the disordered phase) can easily made plausible without calculating the structure factors with formulas. In the case of the latter structural transition, it is even possible to derive a general relationship, as shown in Fig. 7 ▸. The same principle is also readily adaptable to other order–disorder systems, such as that observed in the Cu–Au system. In the latter system, an ordered phase with the *Pm*
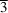
*m* space group (L12, Cu_3_Au type) transforms into a disordered phase with the *Fm*
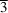
*m* space group (A1, Cu type). Due to space limitations, it is not possible to present all of these systems; however, readers can readily generate these themselves by using the prepared Fourier analysis graphs provided in the supporting information.

### Fourier analysis graphs in structure analysis

**Figure 9:**
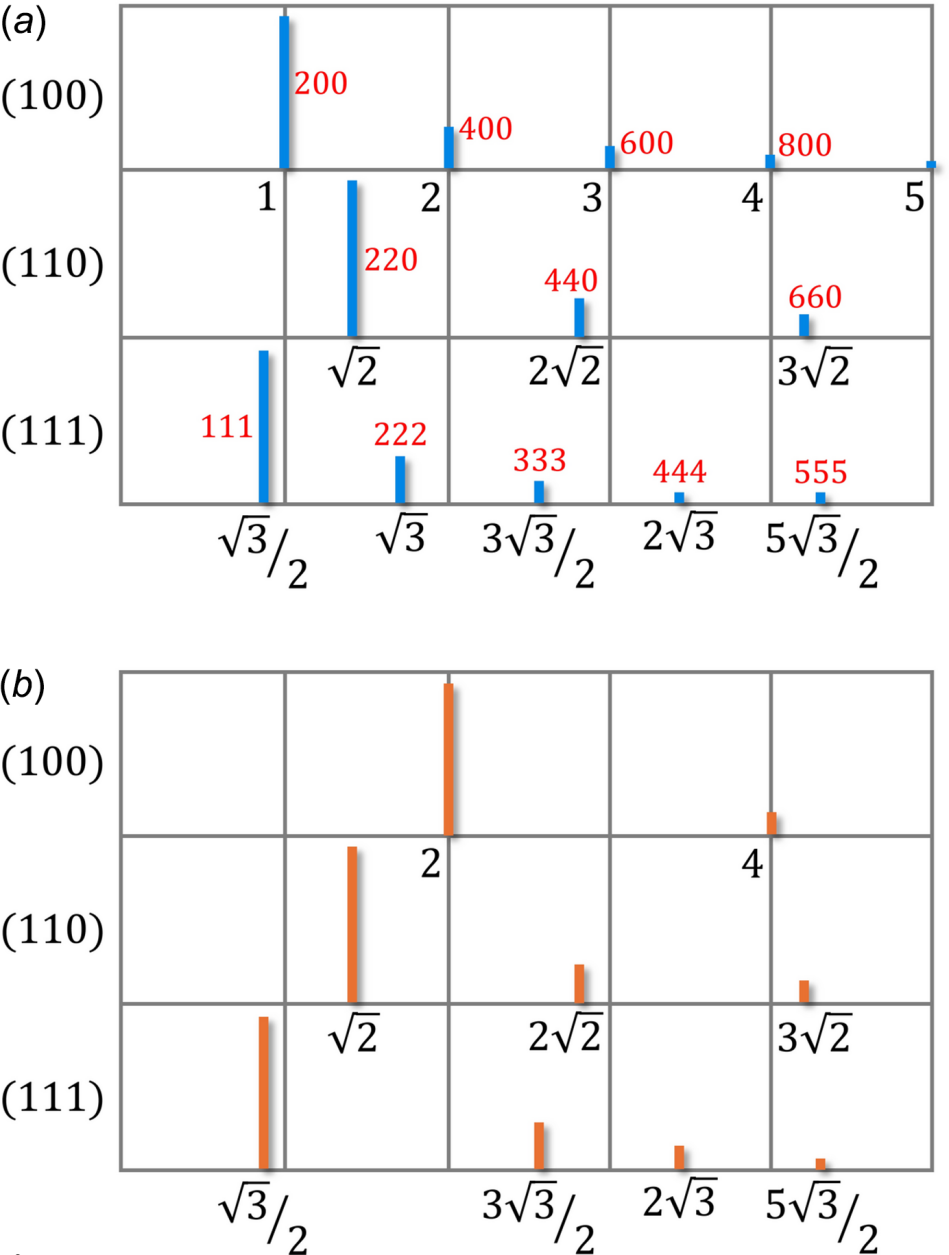
Sequence of reflections for the three prominent crystal faces of a face-centred cubic (fcc) lattice in (*a*) and for diamond in (*b*) redrawn after Bragg & Bragg (1913 ▸). Numbers in black were taken from the original publication and represent the sequence of the reflections for each set of planes as multiples of the first reflection in each series. The latter is always the inverse of the 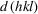 interplanar lattice spacing, with 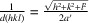, with *a*′ = 1 and *2a*′ = *a* the lattice parameter of the fcc unit cell (see also Bragg, 1913 ▸, p. 270). The numbers in red are the corresponding Laue indices that were added for easier reference to the reflections. It is important to note that, in contrast to the ideal fcc lattice shown in (*a*), the diffraction pattern of diamond in (*b*) lacks the 200, 600, and 222 reflections.

**Figure 10:**
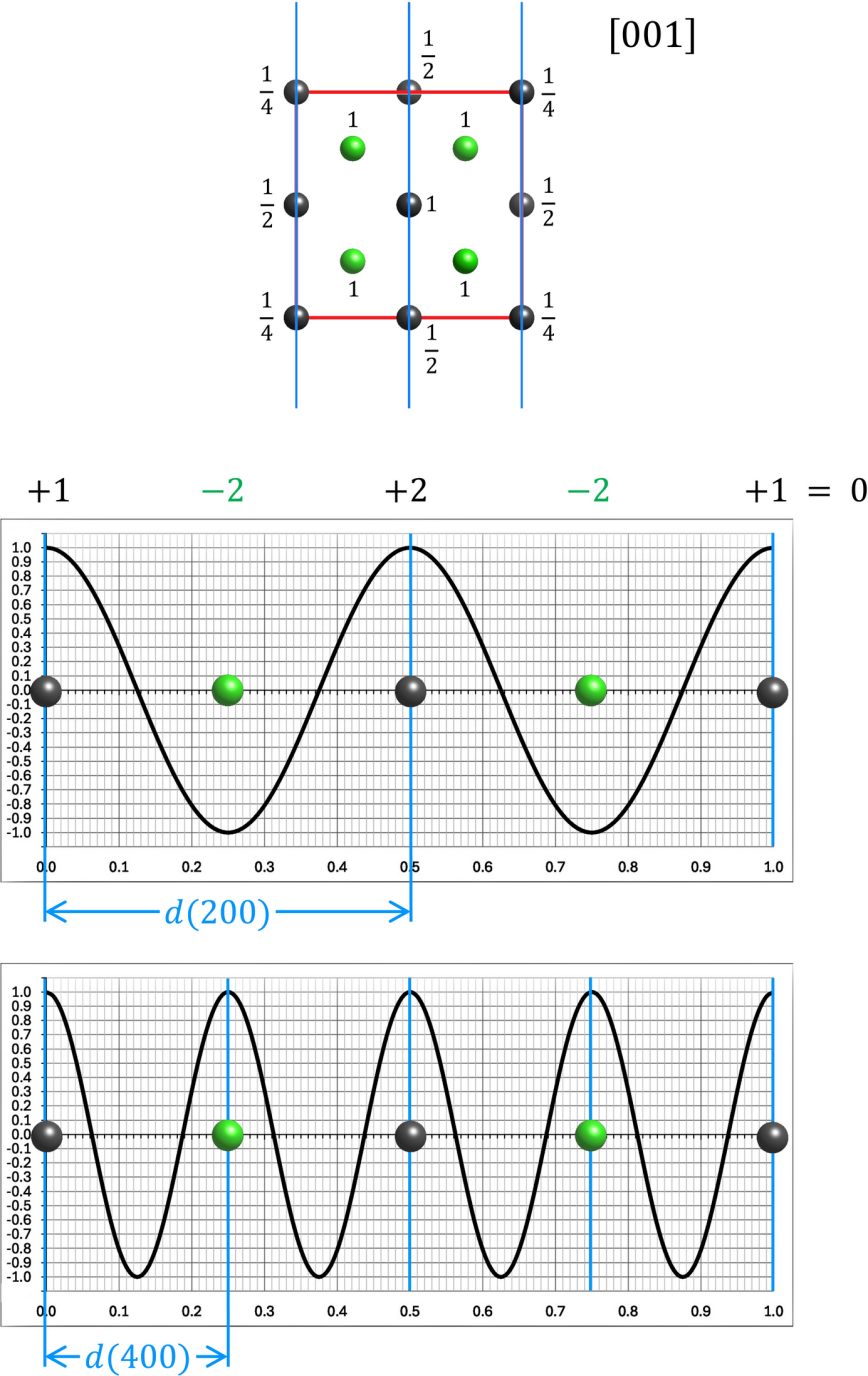
Model for the cubic diamond structure in projection along the [001] direction with corresponding one-dimensional Fourier analysis graphs for the 200 and 400 reflections. Atoms shown in dark grey refer to atoms of the underlying fcc lattice that was assumed by the Braggs in their 1913 ▸ investigation. The green atoms refer to atoms on the tetrahedral sites of the cubic unit cell. The numbers beside the atoms denote their weighting in the numerical summation, *e.g.* atoms on the corners of the unit cell are in fact two atoms projected on top of each other, where each of the atoms is part of 8 unit cells (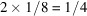), and atoms at the face centres belong to two unit cells and thus are weighted by 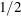. As seen in the 200 graph, the four atoms from the fcc sub-lattice match all with the maxima, so the remaining four atoms must be placed in between at the minima to yield extinction. Acknowledging that the same situation is met if the unit cell is rotated in plane by 90° makes extinction of the 200 (and 020) reflection only possible if the four (green) carbon atoms are placed at *xy* = ¼ ¼; ¼ ¾; ¾ ¼; ¾ ¾ as shown. The corresponding graph for the 400 (or 040) reflection shows that this arrangement results in a positive amplitude for all atoms (all atoms scatter in phase), yielding the strong 400 peak in Fig. 9 ▸(*b*). As this analysis can be repeated for any other equivalent projection of 〈001〉, it must be concluded that the four carbon atoms will be located on the tetrahedral sites of the cubic unit cell. However, a cubic unit cell has eight tetrahedral sites in total, but at this stage only four atoms are remaining to be distributed among them. Thus, a symmetrical distribution can only be obtained by a diagonal distribution of the carbon atoms at each level as shown in Fig. 12 ▸. This also agrees with the model of the projection along [001] in Fig. 10 ▸, where each of the tetrahedral sites is only occupied by one atom (note that in fact two of the tetrahedral sites in three dimensions fall on top of each other in the two-dimensional projection). The model derived thereby is in perfect agreement with the finding that the 200 reflection is extinguished and that 400 is the first strong reflection (see Fig. 9 ▸).

**Figure 11:**
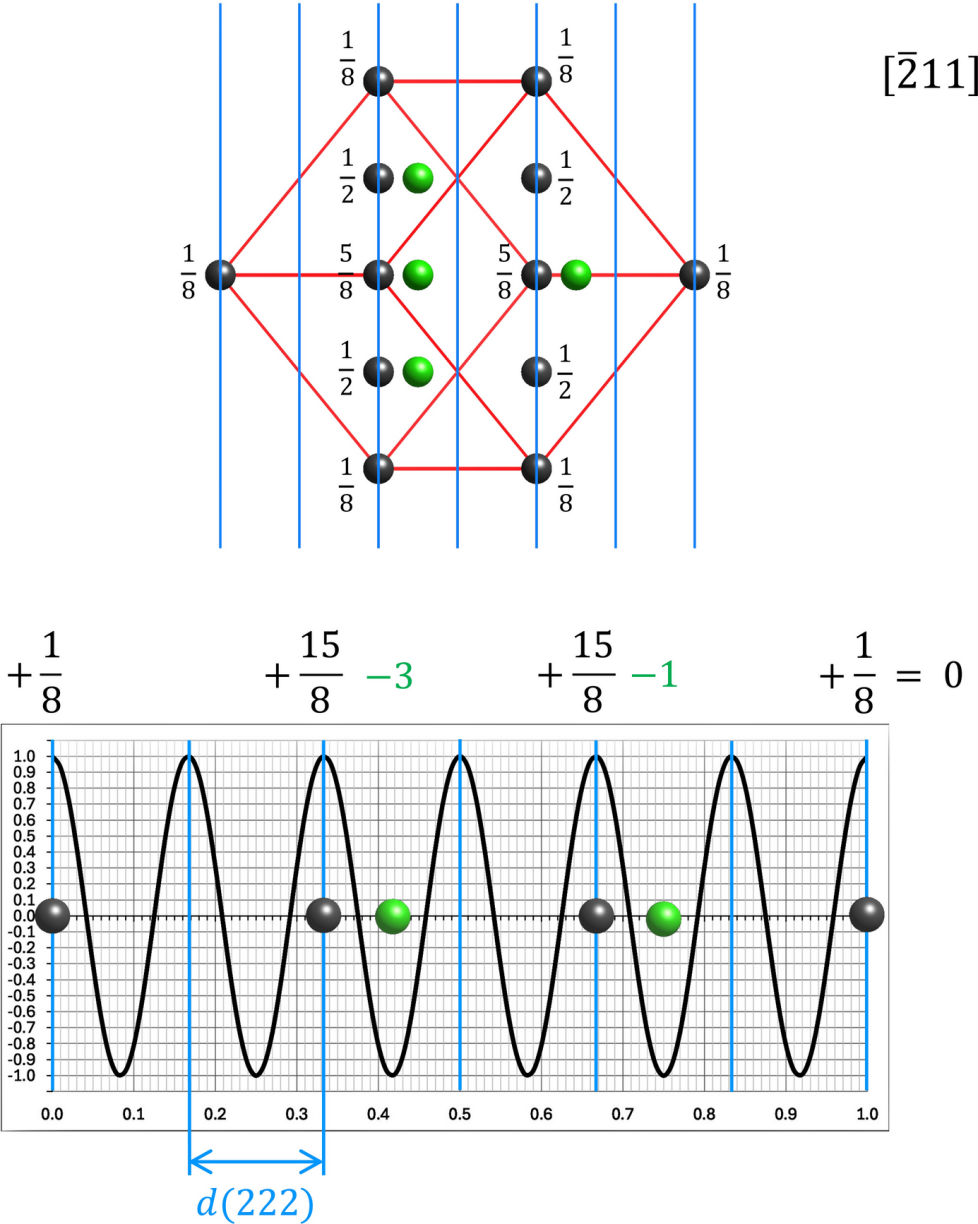
Derived model of the cubic diamond structure in projection along the [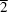
11] direction, together with the graph for the 222 reflection. As can be seen from the contribution of the scattering atoms on the fcc lattice (dark grey) and the half-occupied tetrahedral sites (green atoms), the resulting structure-factor amplitude is zero. This is consistent with the experimental pattern shown in Fig. 9 ▸ and proves the correctness of the determined structure model.

**Figure 12:**
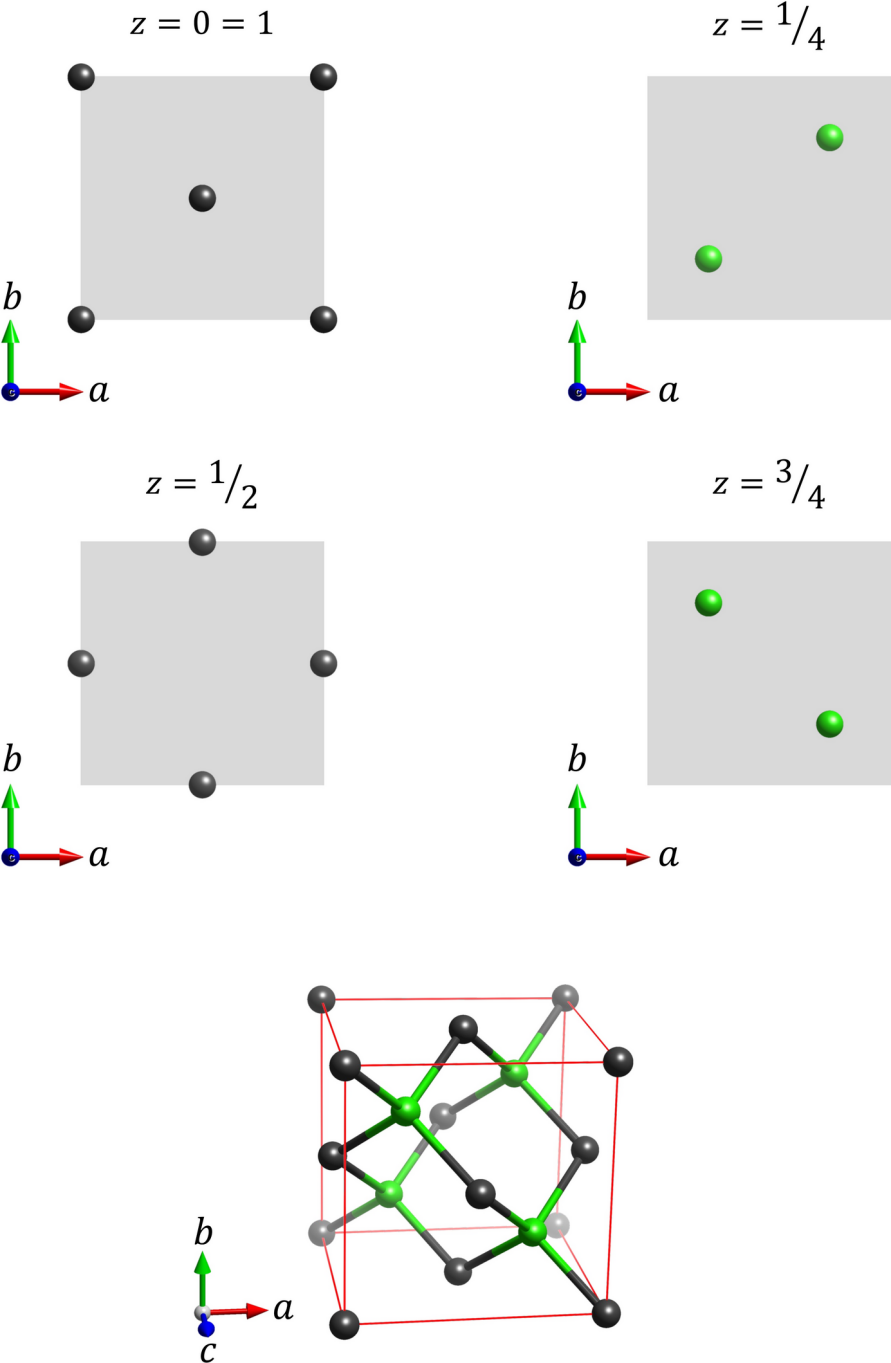
Complete structure model for cubic diamond, deduced from the 200 and 400 reflections (Fig. 10 ▸) via Fourier analysis graphs, and verified by the 222 series of reflections (Fig. 11 ▸). The carbon atoms on the regular fcc lattice (dark grey) and the carbon atoms located at the tetrahedral sites (green) were plotted in different colours to allow easier reference.

The following example follows the footsteps of the Braggs and replays in part the structural analysis of cubic diamond (Bragg & Bragg, 1913 ▸, 1915 ▸, p. 102) using Fourier analysis graphs. Before proceeding with the analysis of the diffraction data, it is first necessary to make a brief comment on the method that was used for recording the diffraction data used here. The Braggs conducted their pioneering structural investigations on small, well-formed single crystals a few millimetres in size, whose crystal faces were indexed by the help of a light reflection goniometer. The X-ray diffractograms were then measured from selected crystal faces, *e.g.* the 100, 110, and the 111 crystal faces, using a proportional counter under reflection (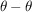) geometry (Bragg & Bragg, 1915 ▸, p. 29; Bragg & Bragg, 1949 ▸, p. 29). This approach allowed the straightforward assignment of the individual diffraction maxima to a specific set of planes as shown in Fig. 9 ▸. Furthermore, the Braggs always took the density of the material into account in their early structural analyses, as this allowed them to verify the size of the unit cell and to determine the number of atoms in the unit cell. The latter enabled them to deduce that the unit cell of diamond contains eight carbon atoms, which was of crucial significance for the correct interpretation of the diffraction pattern as will be seen. From previous studies on other materials with a cubic structure, the diffraction data suggested that a partial lattice of the structure, which covers already four out of the eight carbon atoms, was an fcc lattice. Nevertheless, the task was then to determine the positions of the four remaining carbon atoms within the unit cell. In order to address this question, the focus here is directed towards the two most striking differences in the diffraction pattern of diamond when compared with a conventional fcc lattice. Comparison of the sequence of subsequent diffraction lines for the fcc lattice in Fig. 9 ▸(*a*) and for diamond in Fig. 9 ▸(*b*) reveals absence of the 200, 600, and 222 reflections for diamond. The corresponding graphs for the missing 200 reflection and the first existing reflection in the (100) series, the 400 reflection, are shown in Fig. 10 ▸. As the *B* coefficients of the 200, 400 (and 222) reflections are all zero, these graphs are not shown as they do not contribute to the structure solution. The argument that leads readily to the positions for the four remaining carbon atoms is developed as follows. For the extinction of the 200 reflection, half of the scattering atoms must yield a positive contribution in sum, while the other half must contribute with the same magnitude but with a negative sign to create balance. On the other hand, the 400 reflection must represent a configuration in which all the atoms scatter in phase, thus making the 400 diffraction peak strong [see Fig. 9 ▸(*b*)]. The only arrangement of atoms that is compatible with both conditions (200 extinguished and 400 strong) is to put the remaining four carbon atoms at ¼ and ¾ along the direction of the 200 and 400 cosine Fourier waves (see Fig. 10 ▸). Since the same result will be obtained if the projected unit cell is rotated by 90° around the [001] axis, and the 020 and 040 reflections are considered, the positions of the four carbon (green) atoms in the projection must be at *xy* = 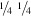; 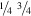; 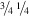; 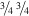 as shown. Since the symmetry is cubic, this argument holds also for any other equivalent projection of *001*, so the four carbon atoms can only be located on the tetrahedral sites of the cubic unit cell. However, since a fcc cell has eight tetrahedral positions, but only four carbon atoms are available for distribution, some of the positions must stay empty. This task can also be resolved from the projection along [001] in Fig. 10 ▸. As demonstrated above, a weighting factor of two is required to balance the (grey) atoms on the fcc sites and consequently, the sites of the (green) atoms can only be half occupied in three dimensions. Moreover, as a symmetry related distribution of the atoms in two and three dimensions is the preferred option, placing two carbon atoms along one diagonal at 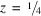 and along the other diagonal at 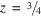 is the only possible solution. This configuration is shown in Fig. 12 ▸ ▸. Note that, in this respect, the structures of diamond and the zinc blend are very closely related to each other. The model derived here corresponds perfectly with the observation that the 200 reflection is extinguished and the 400 reflection is the first that appears for the (100) crystal face (see Fig. 10 ▸). Further proof for the correctness of the model is also provided by the fact that the 222 reflection is absent. This is shown in Fig. 11 ▸ for the derived model in projection along [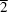
11]. As seen for the corresponding 222 reflection, the (positive) contributions of the carbon atoms on the regular fcc lattice balance the (negative) contributions of the carbon atoms on the tetrahedral sites, so that the structure factor amplitude becomes zero.

### Discussion and conclusion

The one-dimensional Fourier analysis method presented offers an alternative to the conventional equation-based teaching of the structure factor, as it provides a simple graphical approach of linking the diffraction pattern of a crystalline material with its underlying atomic structure. The used methodology of discrete Fourier analysis employs sine and cosine plots for different orders of reflection (see supporting information), which are superimposed by the one-dimensional structure obtained by projection. At the atomic positions, the values of the cosine and sine functions are multiplied by the appropriately weighted atomic scattering amplitudes, and the resulting contributions are summed. In this way, the *A* and *B* coefficients of the structure factor are obtained graphically, an approach that students often find more intuitive than the abstract summation represented by equations 2 and 3. In this context, it is important to emphasize that the method is based on discrete Fourier analysis and is distinct from the one- and two-dimensional structure-factor graphs (Bragg–Lipson charts) that were sometimes employed in the pre-era of Direct Methods for structure analysis (see Stout & Jensen, 1989 ▸, p. 430; Lipson & Cochran, 1966 ▸). As demonstrated by the examples given, reducing the structure to one dimension does not yield practical limitations, and different atom types and site-occupation numbers can be readily incorporated into the analysis through weighting factors. Moreover, it has been demonstrated that the approach is also applicable to simple cases of structure analysis. Nonetheless, it should be clear that this approach is primarily intended as a teaching tool and was not developed to compete with established methods for crystal structure analysis. Thus, its strength lies more in the development of a conceptual understanding for already known structures as was demonstrated by several examples.

## Supplementary Material

Suppl. Material: ready for use plots of A(h) and B(h). DOI: 10.1107/S2056989026000745/oi2031sup2.pdf

## Figures and Tables

**Figure 8:**
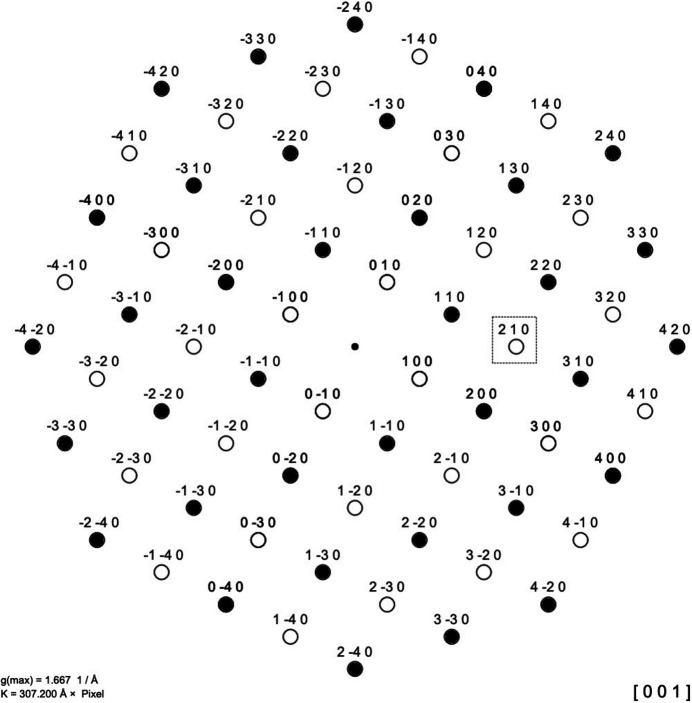
Schematic diffraction pattern of the caesium chloride (CsCl-type) structure in projection along the [001] direction. As explained in the caption of Fig. 7 ▸ referring to the boxed 210 reflection, the open circles represent weak diffraction spots if the scattering powers of the *A* and *B* ions in the ordered structure are different. If complete disorder takes place, so that the scattering power of the *A* and *B* atoms is in balance, the weak reflections will disappear and the remaining pattern will be that of a simple bcc structure, represented by the filled diffraction spots.

**Figure 13:**
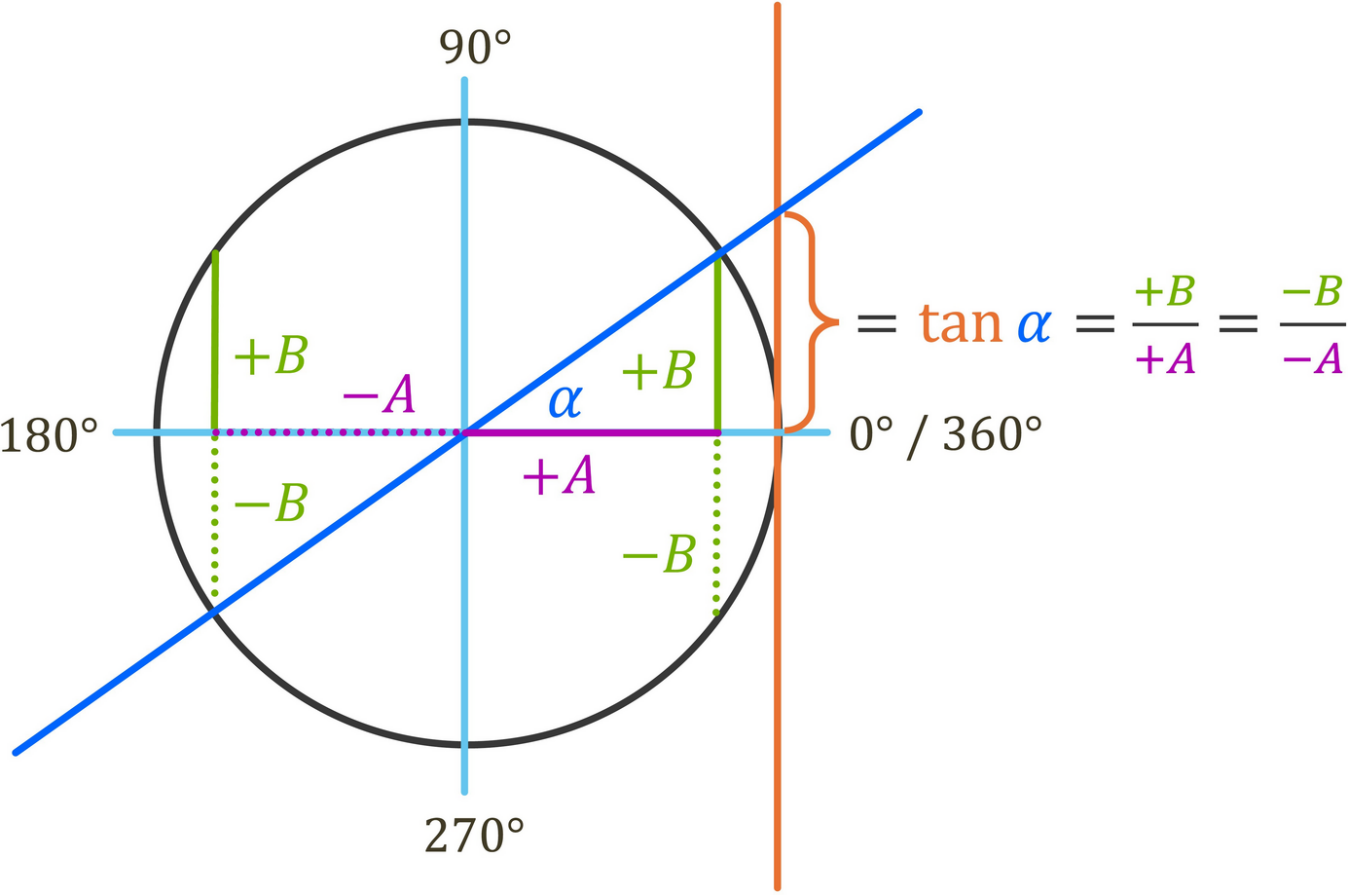
Representation of the structure-factor components *A* and *B* of different signs within the complex plane (Argand plot). For numerical calculations using equation 4, it is important to note that there exists an ambiguity regarding the true value of the phase angle 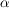 of the structure factor. This is because the slope determined from equation 4 is identical if the signs of *A* and *B* are both the same (as shown in the above example) or if they are different, which yields a negative slope. As the true value for the phase angle 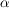 is dependent on the signs of *A* and *B*, it may require a correction. So, for example, if *A* is positive and *B* is negative, the permitted phase angle must be within 270° (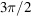) and 360° (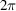). The necessary corrections are summarized in the lookup table in Table 1 ▸, together with an example for each.

**Table 1:** The phase angle 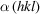 of the structure factor, as determined from equation 4, may require a correction because the phase angle is not only dependent on the absolute values of *A* and *B*, but also on their signs (see Fig. 13 ▸). The corresponding rules for correcting the phase angle are given in the subsequent table, where is the angle initially obtained from equation 4

*A*	*B*	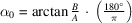	Examples for magnitudes |*A*| = |*B*|
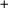	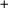	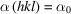	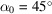 : no correction
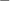	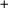	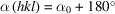	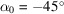 : 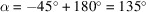
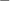	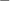		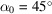 : 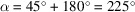
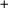	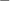	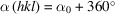	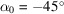 : 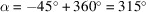

## Appendix A Lookup table for correction of the structure factor phases

Fig. 13 ▸ shows a representation of the structure factor components (Argand plot), and Table 1 ▸ gives corrections for the phase angle.
