# Supplementary material for: Graphical Fourier-coefficient analysis as a paper-based method for teaching structure factors
Source: Acta Crystallogr E Crystallogr Commun. 2026 Jan 29;82(Pt 2):235–43. doi: 10.1107/S2056989026000745 (PMC12874239; doi:10.1107/S2056989026000745)
Supplement: Supplementary file 1 [file e-82-00235-sup2.pdf]

Graphical Fourier-coefficient analysis as a paperbased method for teaching structure factors

T.E. Weirich (2026). Acta Cryst. E82 https://doi.org/10.1107/S2056989026000745.

Supporting Material

h = 1

$Scale\colon\ x_j\ [mm] = \ x_j \cdot 150\ mm$

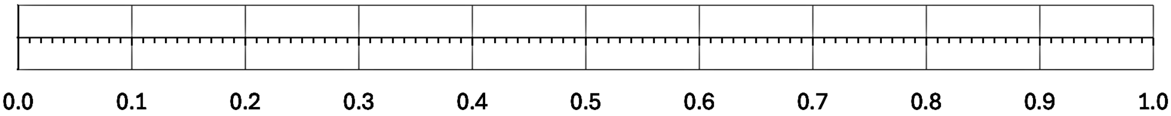

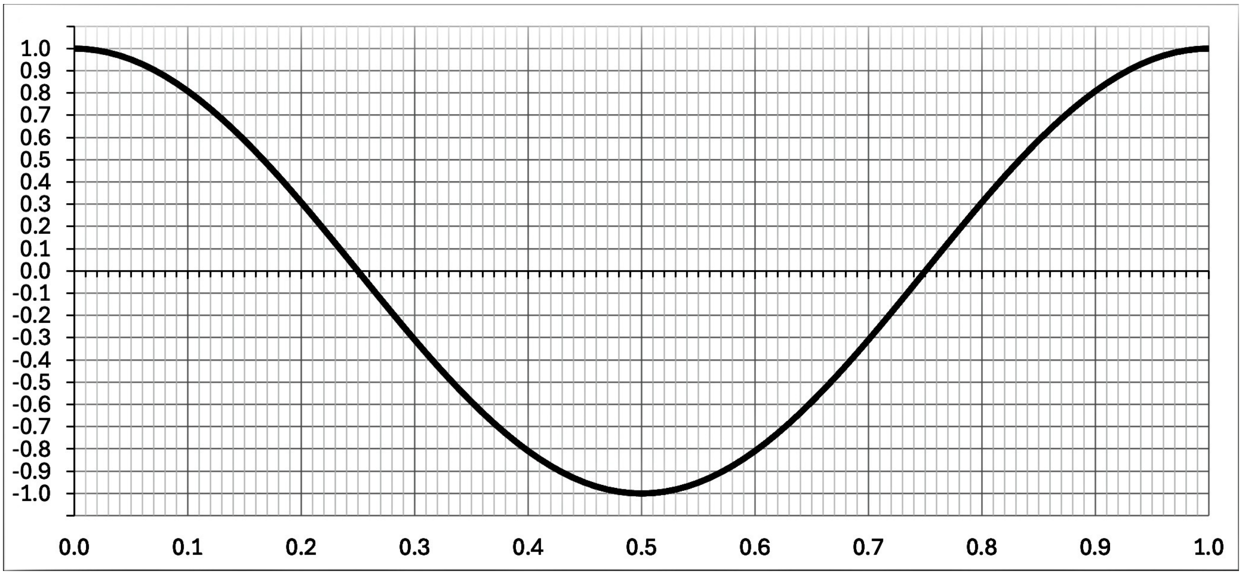

A

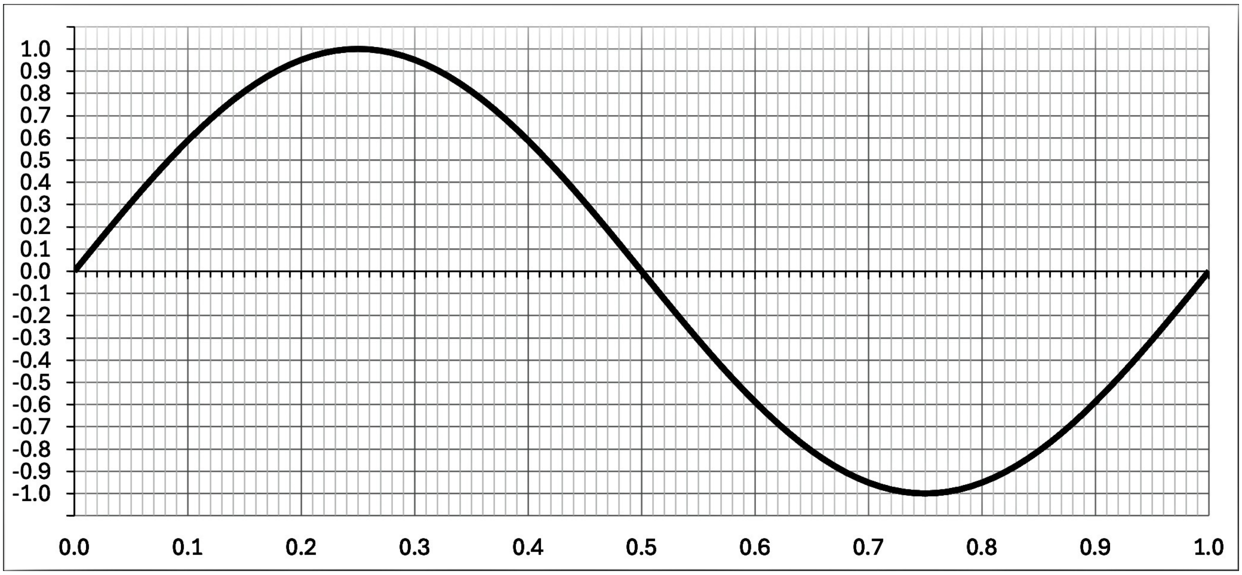

B

h = 2

$Scale\colon\ x_j\ [mm] =\ x_j \cdot 150\ mm$

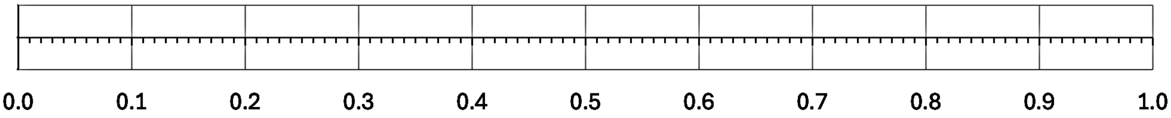

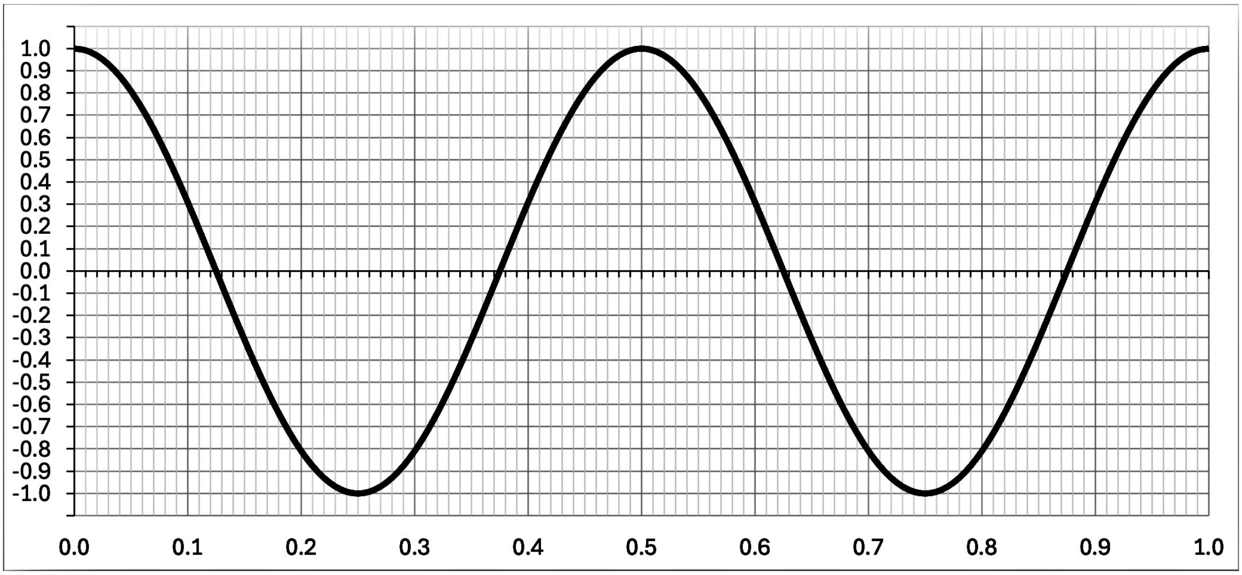

A

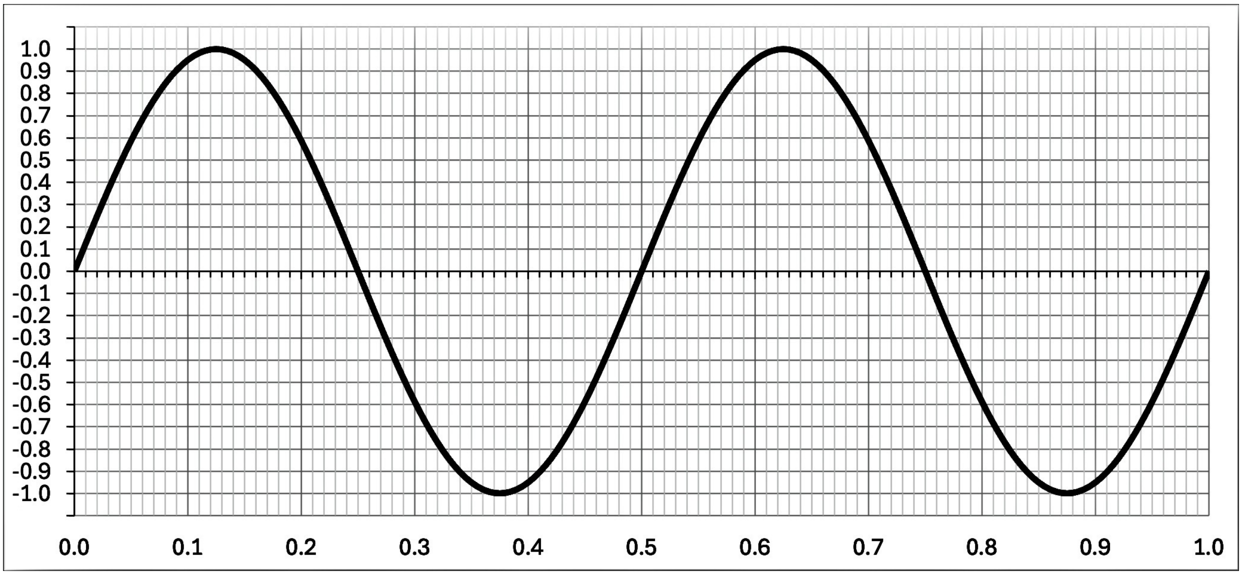

B

h = 3

$Scale: \ x_j \ [mm] = \ x_j \cdot 150 \ mm$

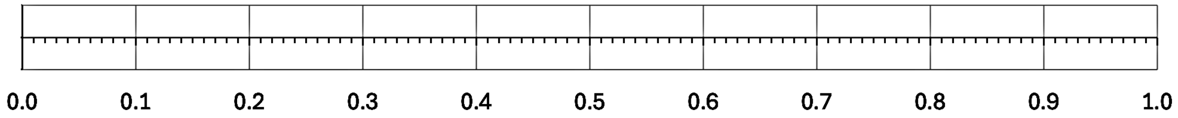

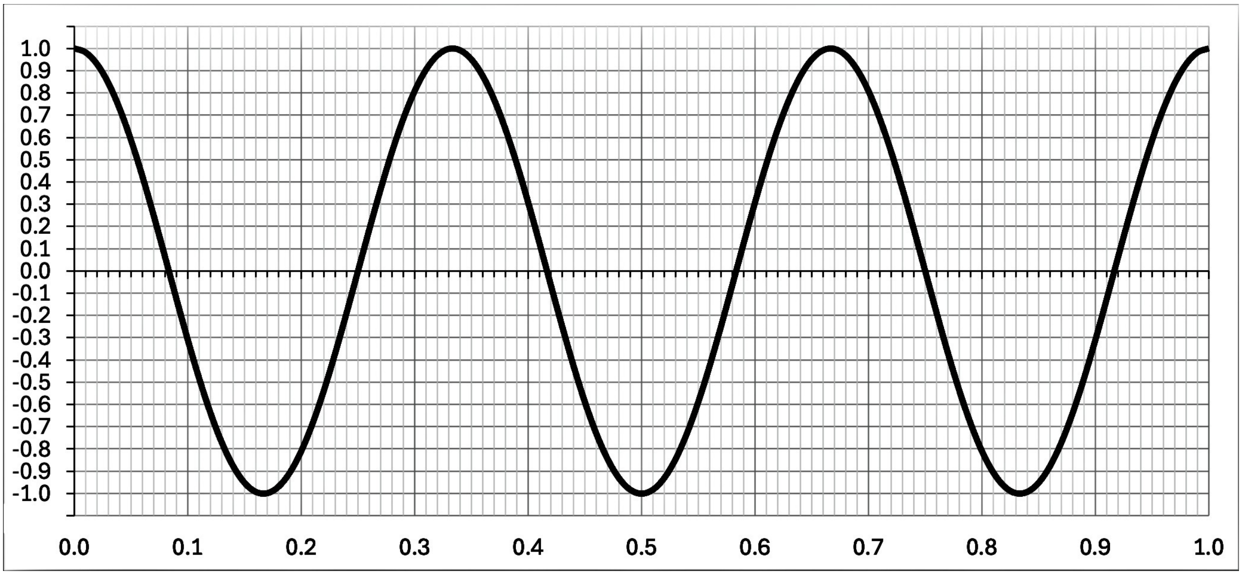

A

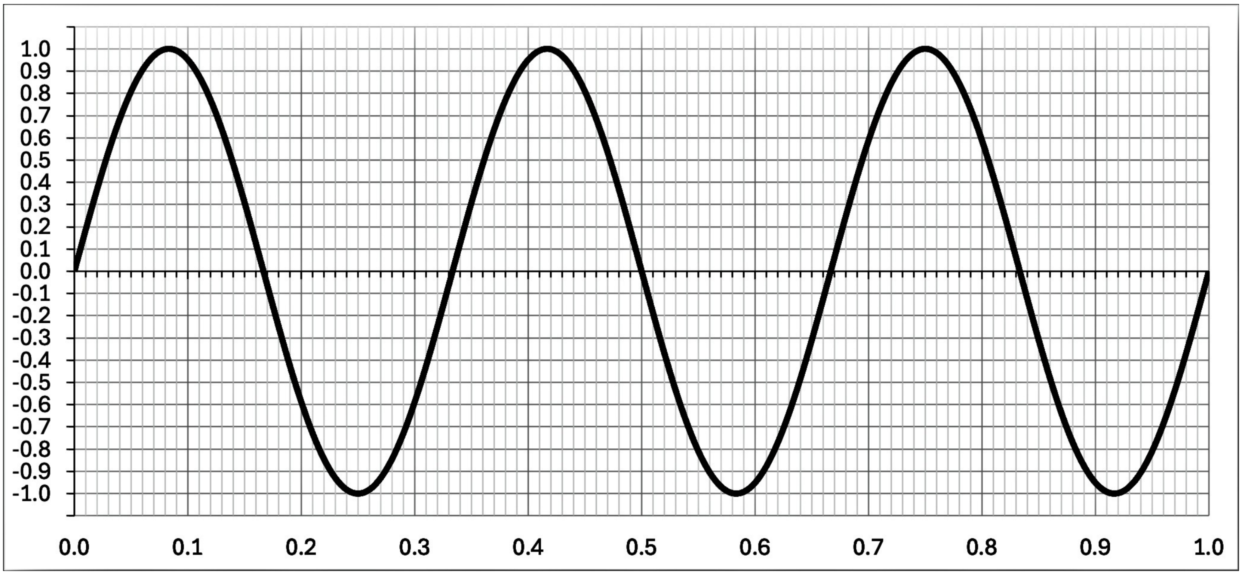

B

h = 4

$Scale\colon\ x_j\ [mm] =\ x_j \cdot 150\ mm$

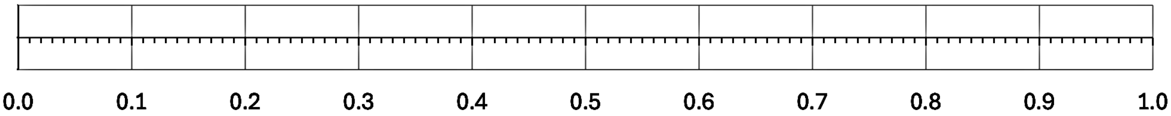

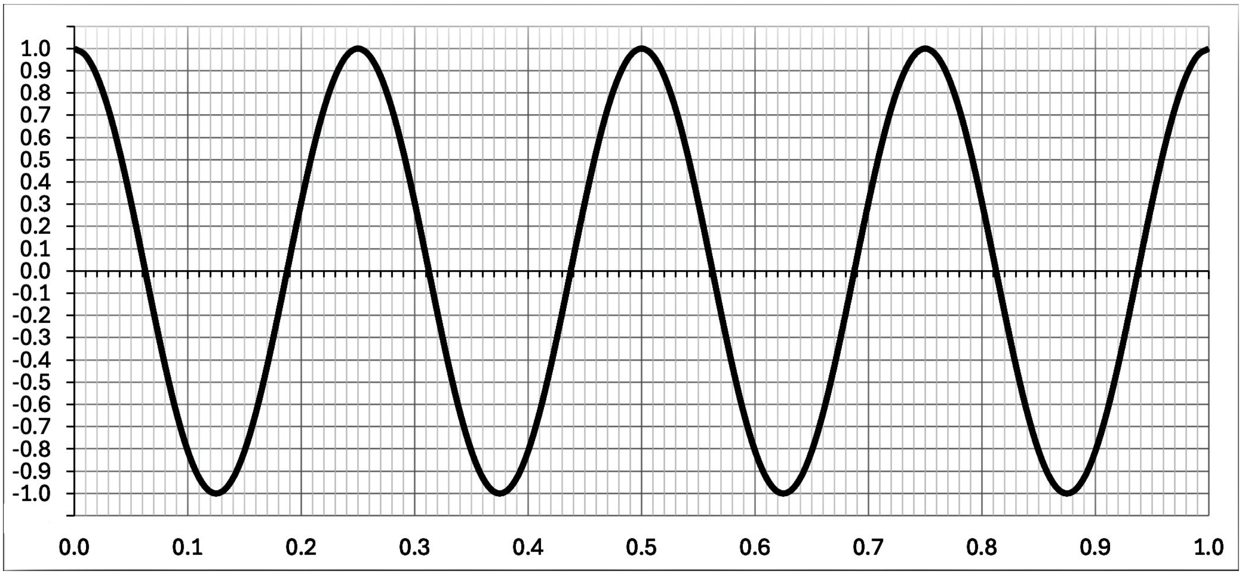

A

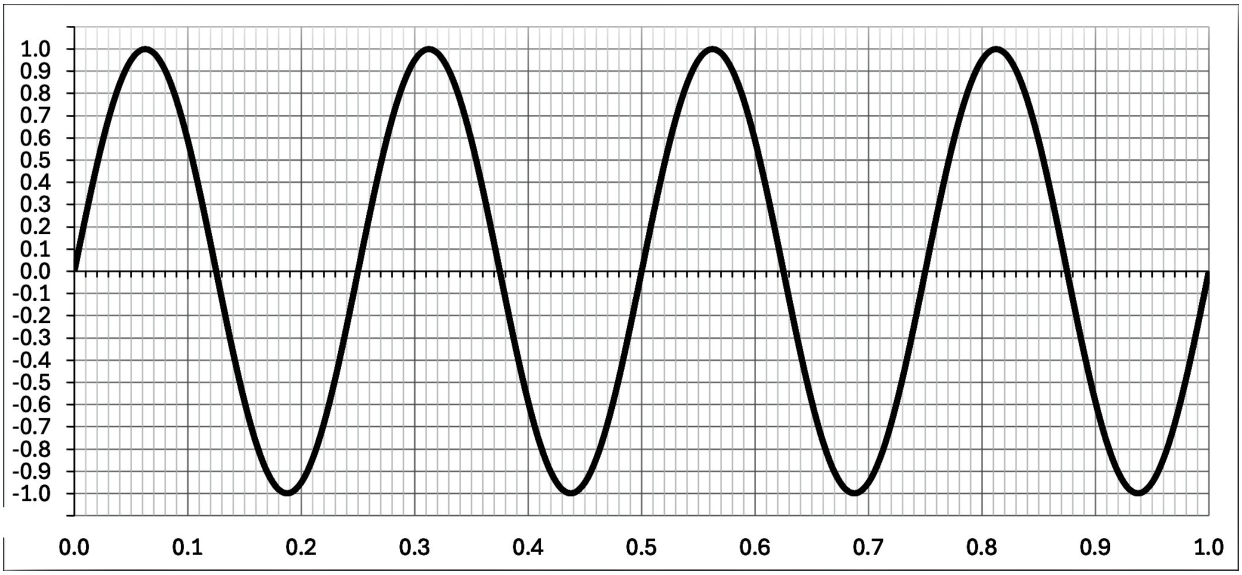

B

T.E. Weirich (2026). Acta Cryst. E82, https://doi.org/10.1107/S2056989026000745

$h = 5$

$Scale: \; x_j \, [mm] = \; x_j \cdot 150 \, mm$

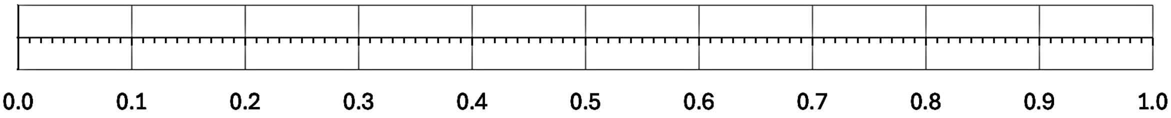

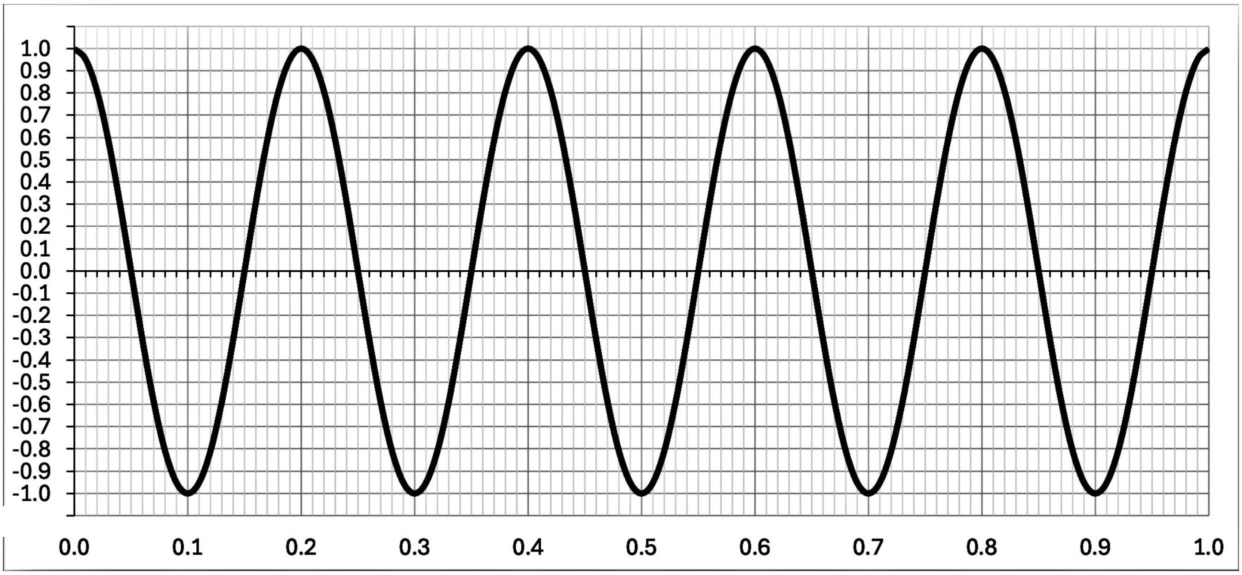

A

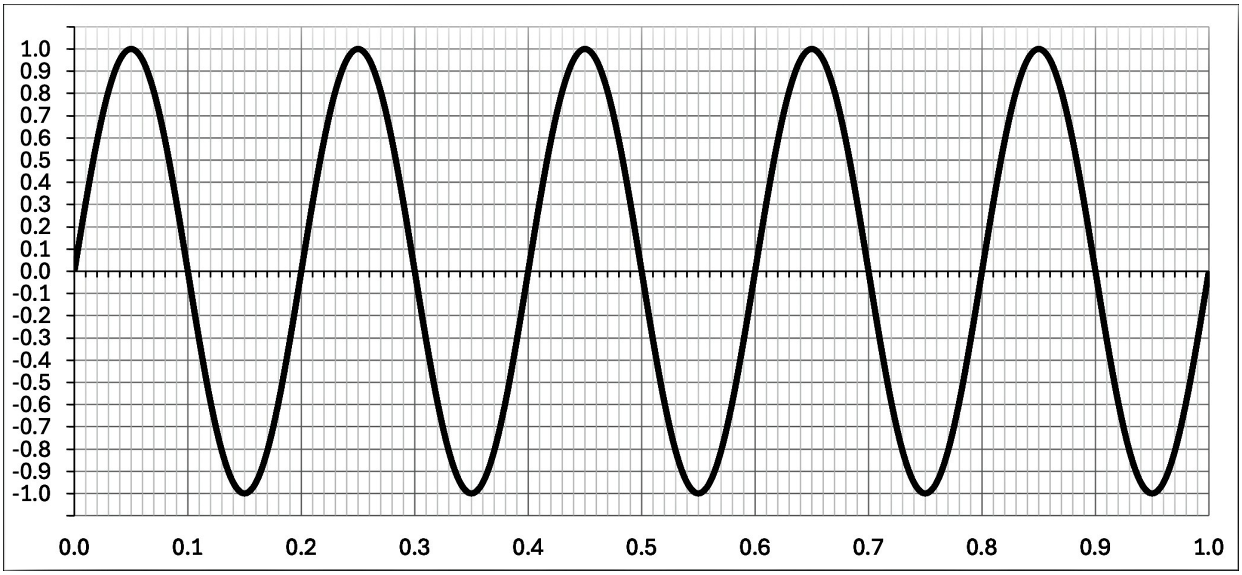

B

T.E. Weirich (2026). Acta Cryst. E82, https://doi.org/10.1107/S2056989026000745

h = 6

$Scale\colon\ x_j\ [mm] =\ x_j \cdot 150\ mm$

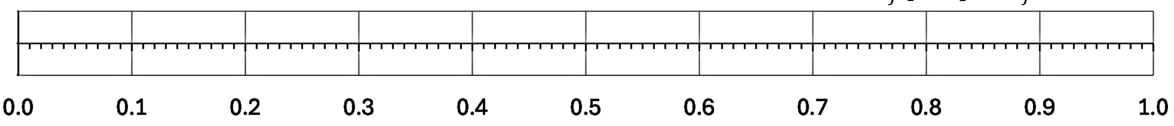

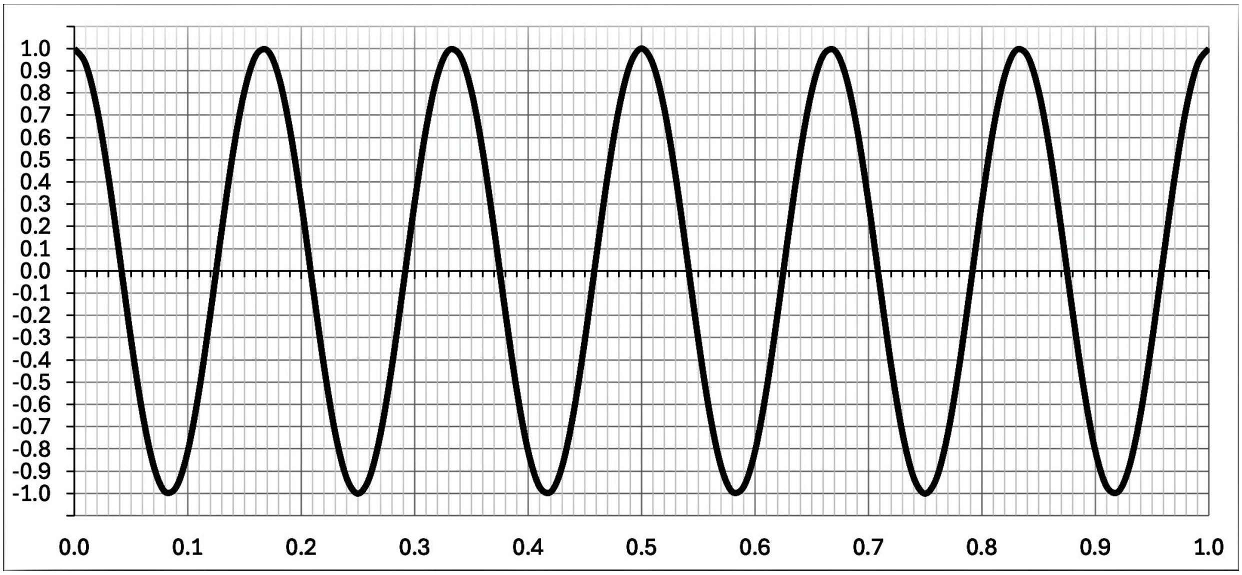

A

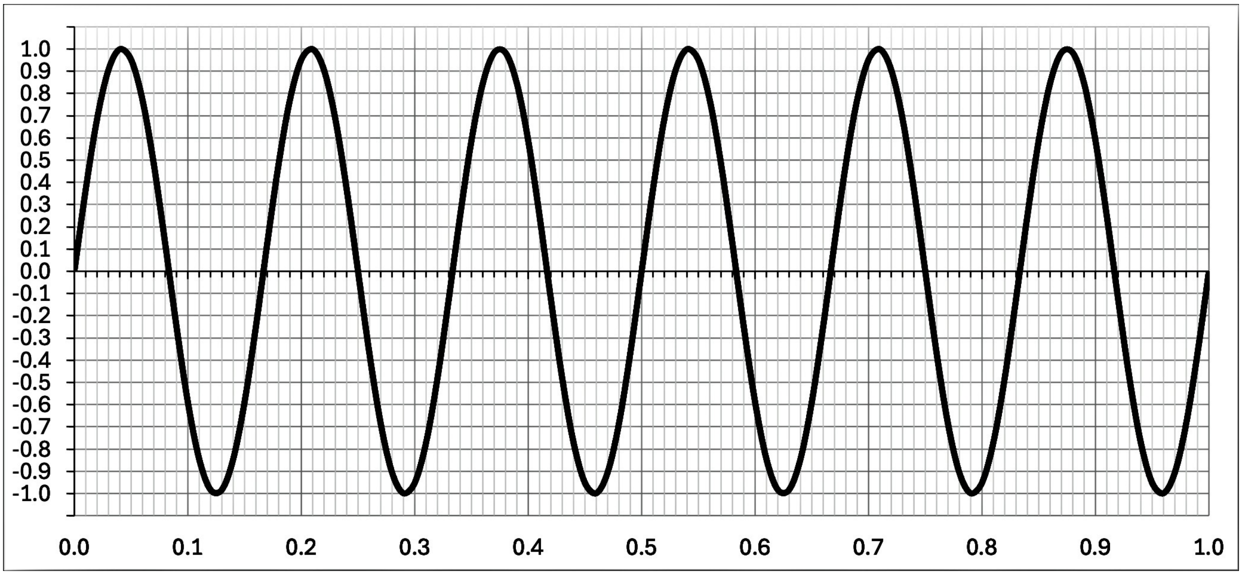

B

T.E. Weirich (2026). Acta Cryst. E82, https://doi.org/10.1107/S2056989026000745

h = 7

$Scale\colon\ x_j\ [mm] =\ x_j \cdot 150\ mm$

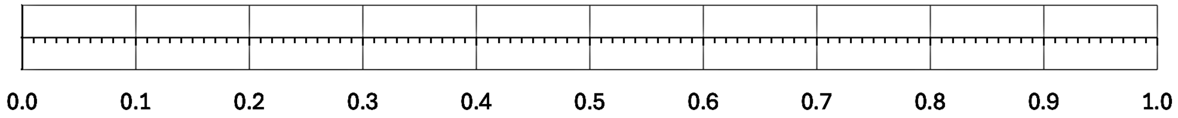

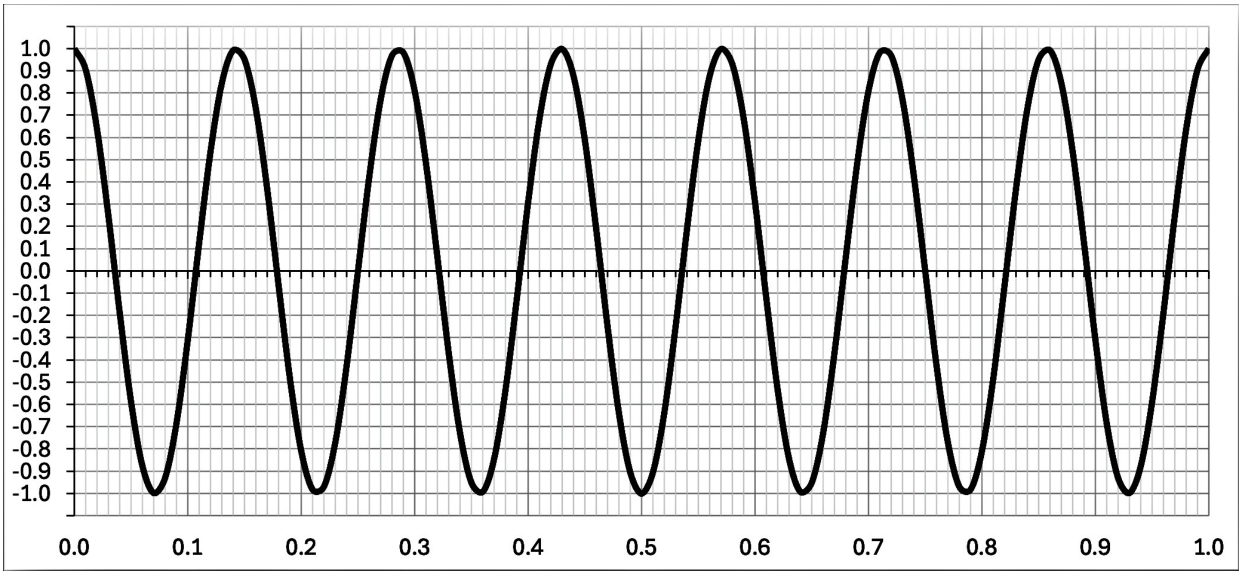

A

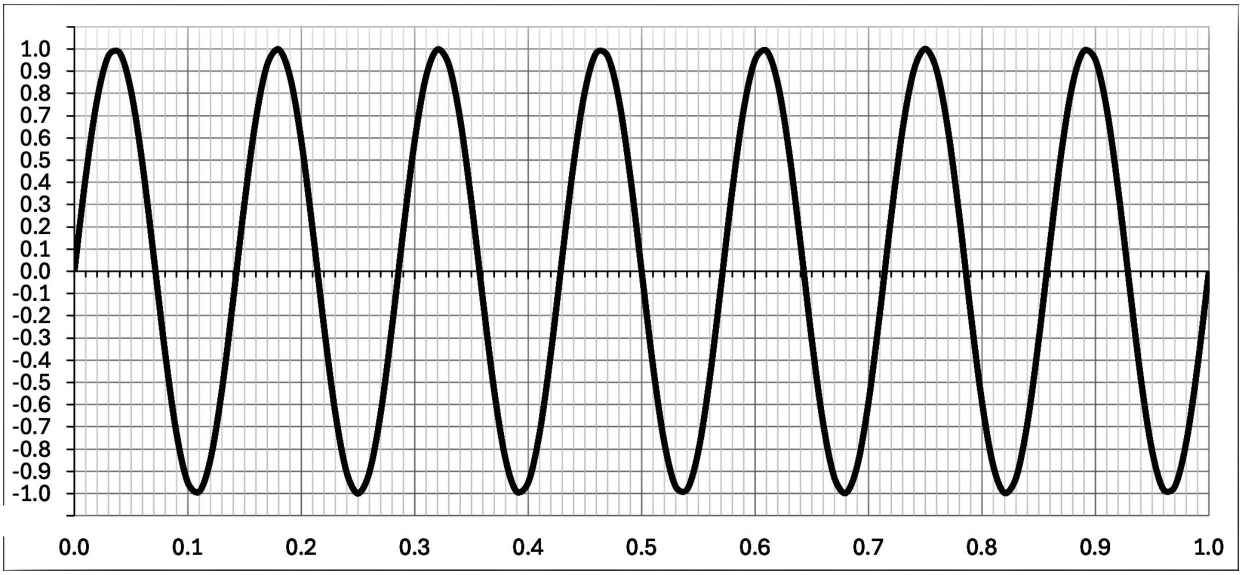

B

h = 8

$Scale\colon\ x_j\ [mm] =\ x_j \cdot 150\ mm$

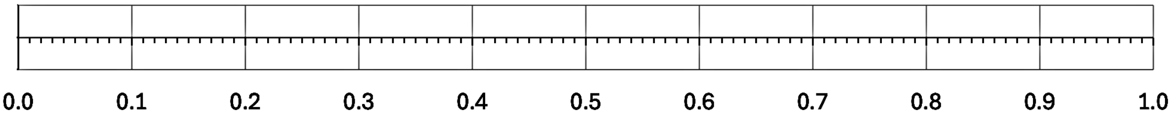

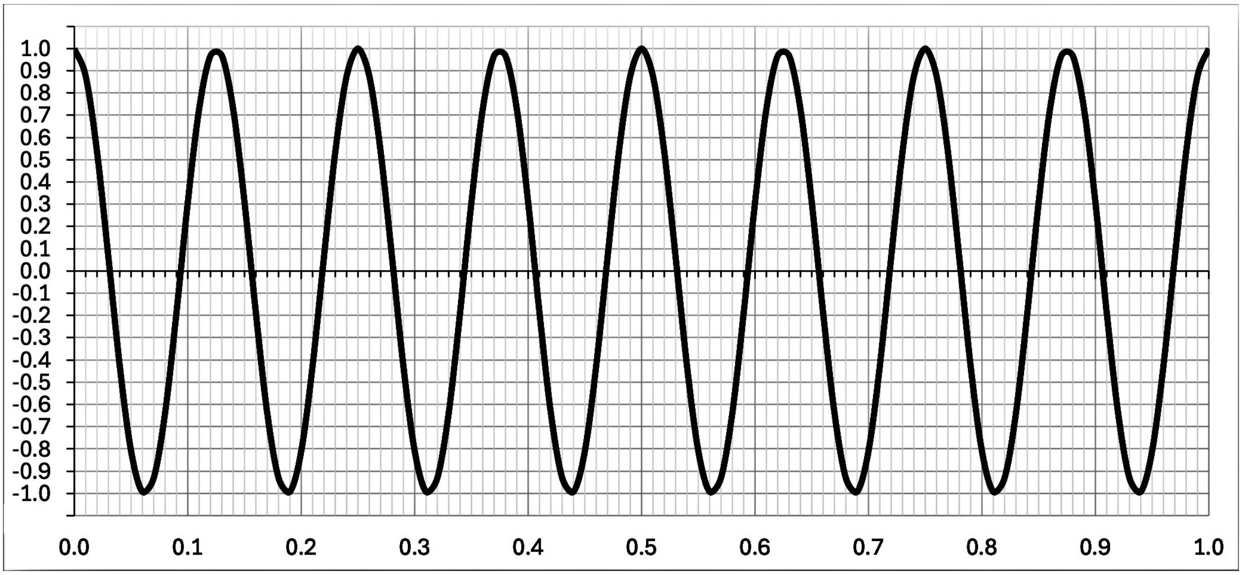

A

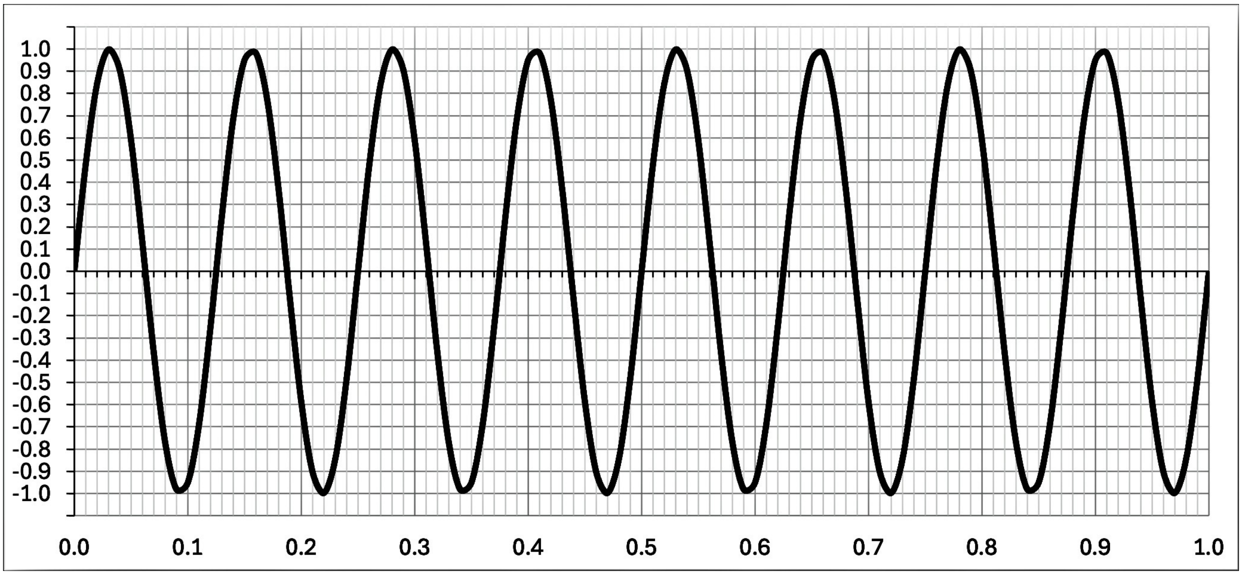

B

h = 9

$Scale\colon\ x_j\ [mm] =\ x_j \cdot 150\ mm$

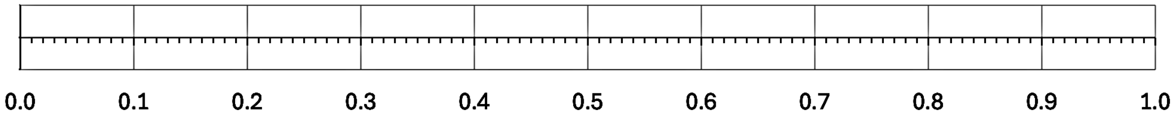

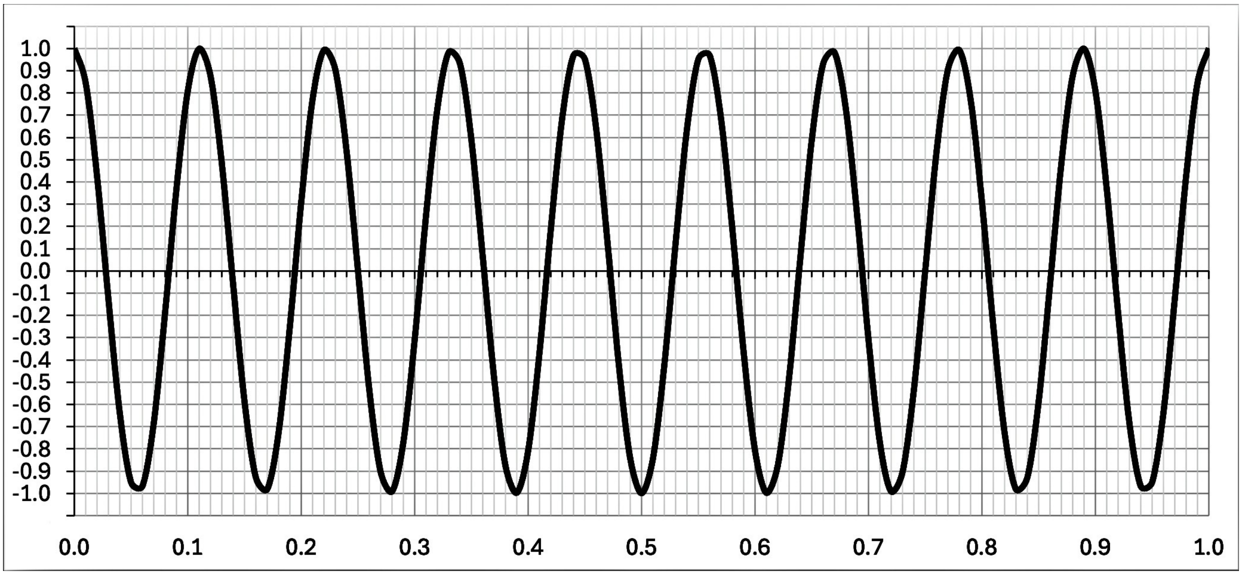

A

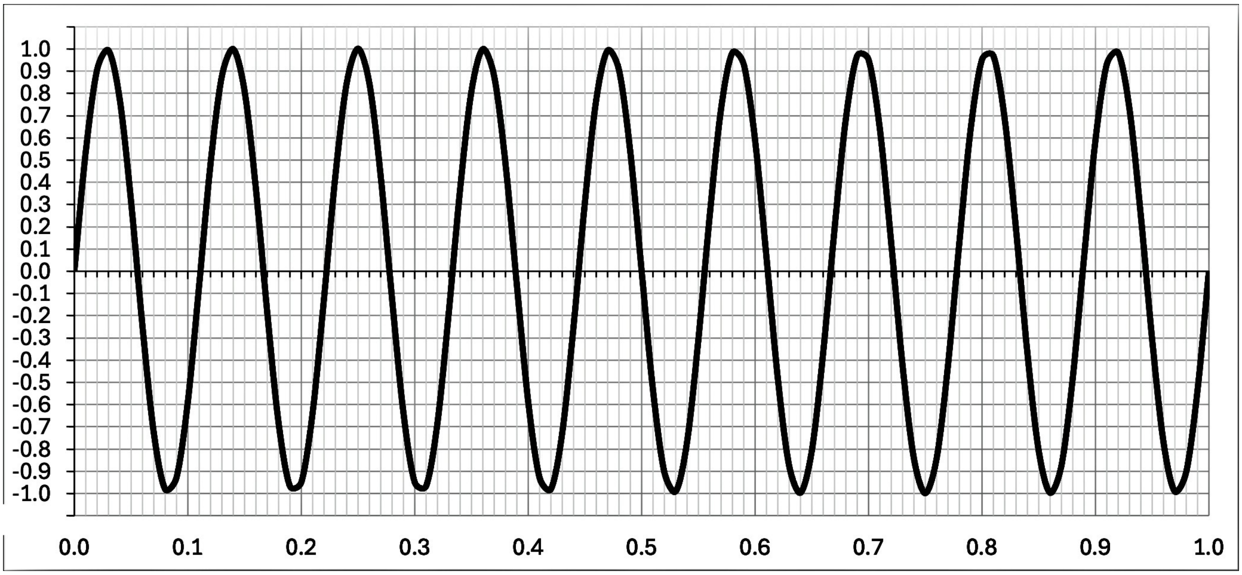

B

h = 10

$Scale{:}\ x_j\ [mm] =\ x_j \cdot 150\ mm$

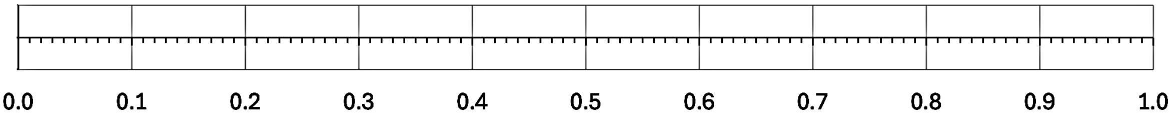

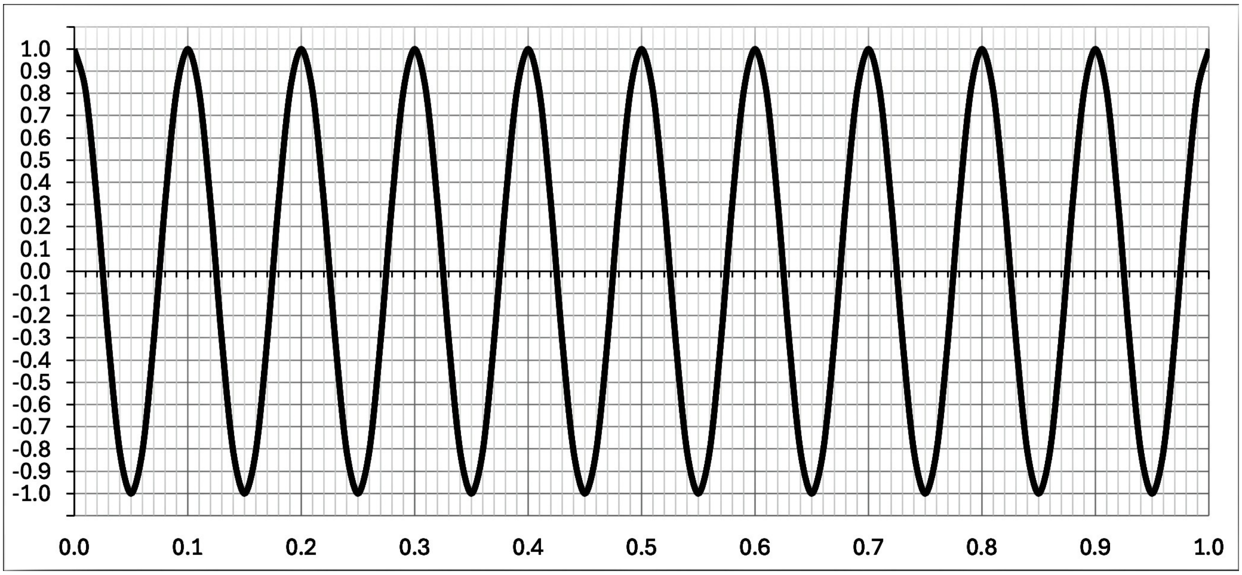

A

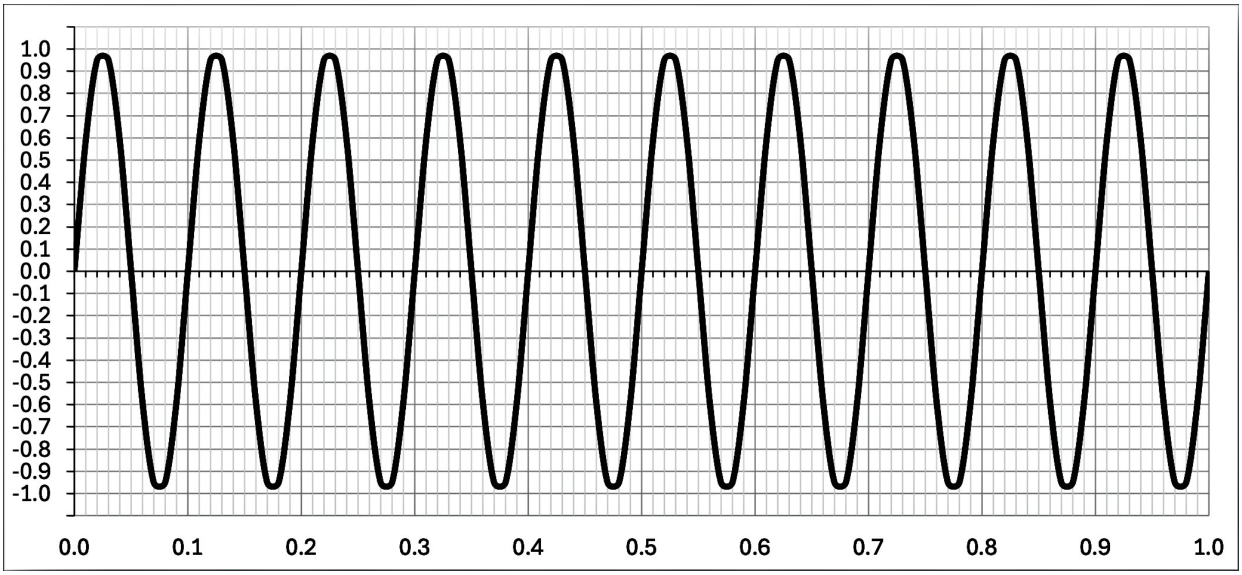

B
